# Molecular Principles of Gating Proton Transport in the Antiporter Modules of Respiratory Complex I

**DOI:** 10.1021/jacs.6c05956

**Published:** 2026-05-08

**Authors:** Sofia Badolato, Sarah C. Rossmann, Joana Pereira, Hyunho Kim, Ville R. I. Kaila

**Affiliations:** Department of Biochemistry and Biophysics, 7675 Stockholm University, Stockholm 10691, Sweden

## Abstract

The respiratory Complex I is a highly intricate redox-driven proton pump that powers oxidative phosphorylation across all domains of life. Yet, despite major efforts, its long-range energy transduction principles remain much debated. Here, we study the molecular principles of proton transport by engineering the antiporter modules of Complex I. By combining directed mutagenesis with time-resolved spectroscopy and molecular dynamics (MD) simulations, we identify conserved residues along the proton channels that control the rate of proton transfer across proteoliposome membranes. The antiporter modules catalyze this tightly regulated proton transport by transient water wires that follow intrinsic electric fields along the proton channels. Based on MD simulations, we identify conserved gating sites, established by nonpolar residues, which modulate the hydration and electric field effects underlying the proton transport upon mutation. On a general level, our findings highlight how the modular energy-transduction machinery of Complex I employs a combination of electrostatic and conformational coupling principles to catalyze long-range proton transport, with distinct similarities to other enzymes.

## Graphical abstract

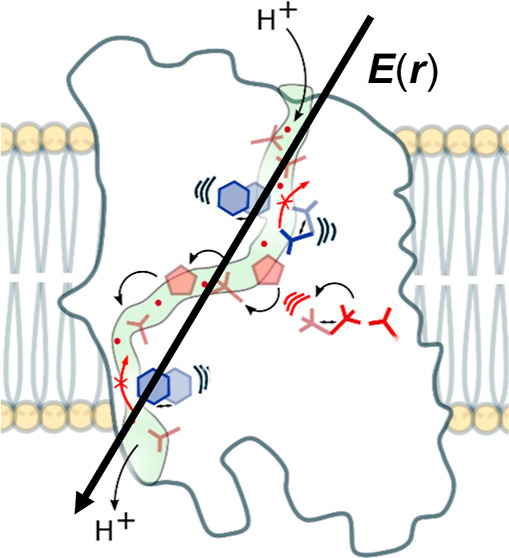

## Introduction

Complex I (NADH/ubiquinone oxidoreductase) is a redox-driven proton pump that powers cellular respiration in mitochondria and many bacteria,^1–5^ using a fully reversible long-range proton-coupled electron transfer (PCET) process that extends across a remarkable distance of >300 Å. The process is powered by electron transfer between nicotine amide adenine dinucleotide (NADH) and quinone (Q) in the hydrophilic domain of Complex I that drives proton pumping across its 200 Å wide membrane domain (Figure 1A). Yet, despite detailed experimental and computational efforts over the past decades,^6–13^ the long-range energy-transduction mechanism of Complex I remains unsolved and much debated.

**Figure 1:**
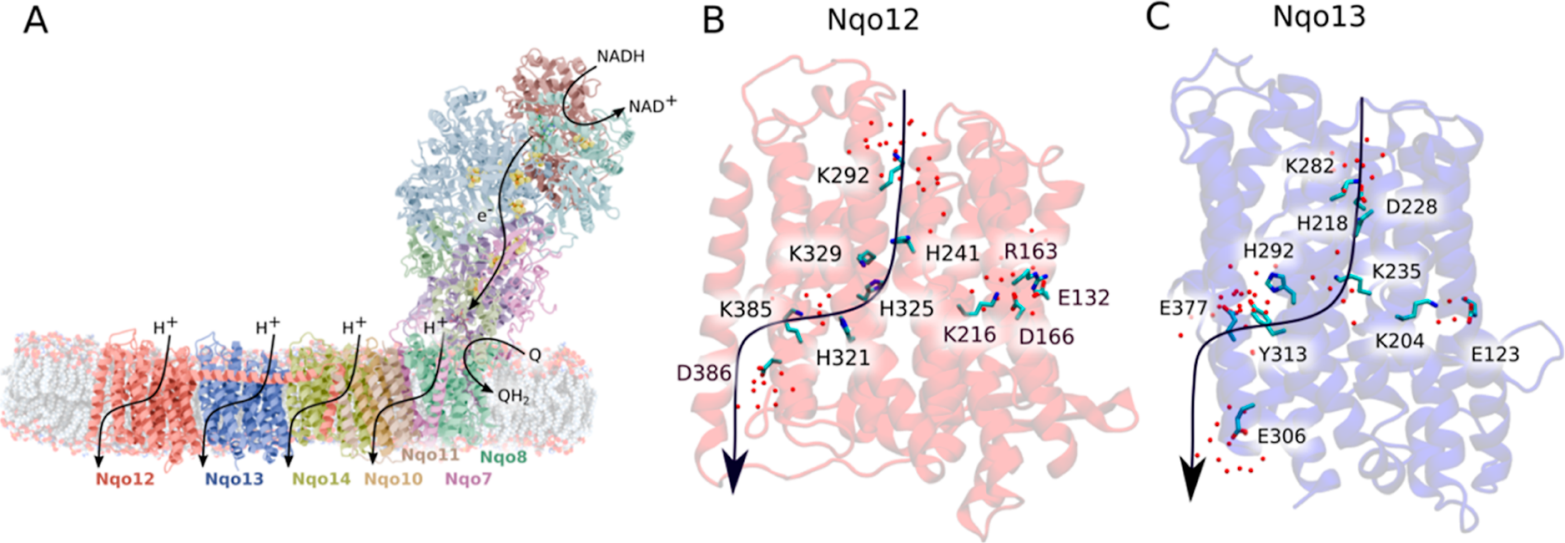
Structure and function of Complex I. (A) NADH-driven quinone reduction powers proton transport across the antiporter modules. (B) The terminal (Nqo12) and (C) the penultimate (Nqo13) antiporter modules establish putative proton channels across the membrane.

For each NADH oxidation step, Complex I pumps four protons across its membrane domain. It has been suggested^8,14,16,17^ , but *cf*. ref 15 that each antiporter-like module, Nqo12, Nqo13, and Nqo14 together with Nqo7/8/10/11 (Figure 1A), forms S-shaped proton wires, comprising water molecules and conserved titratable residues that are responsible for the proton translocation (Figure 1B,C). This water structure is in part also supported by recent cryo-electron microscopy (cryo-EM) data, particularly along the central hydrophilic axis, although a complete connectivity across the positively charged side (P-side) of the membrane has so far been experimentally observed only in the terminal Nqo12 subunit (Figure 1B).^7,18^ The functional relevance of the conserved residues along the pathways is indirectly supported by mutagenesis experiments,^19,20^ where substitutions result in the partial or complete blocking of the coupled proton–electron transfer activity. However, the tight coupling between electron transfer activity in the hydrophilic domain and the proton pumping in the membrane domain renders the investigation of functionally relevant residues highly challenging, as mutation of central residues blocks both activities.^9,10,19–22^

We have suggested^10,23,24^ that the redox-driven proton pumping in Complex I takes place by propagation of a “protonation wave” along the membrane module, triggered by the quinol formation at the interface of the hydrophilic and membrane domains.^25–28^ Each antiporter-like subunit forms an S-shaped proton wire, establishing transient hydrogen-bonded connections across the membrane^14,29^ (*cf*. also refs 15 and 30), while conformational changes in conserved ion-pairs within each antiporter module stimulate proton transfer across the lateral hydrophilic axis and result in a stepwise propagation of the protonation reactions toward the terminal Nqo12 subunit. Proton release from the P-side output channel of Nqo12 was suggested to result in a “back-wave”, involving stepwise proton uptake along the N-side input channel and conformational changes in the ion pairs that eventually release the quinol species, bound at the second membrane-bound binding site, supported by both molecular simulations^31–33^ and cryo-EM^5,34^ data.

In contrast to the wave propagation model, where each antiporter-like subunit pumps one proton, Sazanov and co-workers proposed^7,18^ that half of the antiporter-like subunits (Nqo12 and Nqo13) are involved in the proton uptake from the N-side, but all *four* protons are released to the P-side through the terminal subunit Nqo12, mediated via lateral proton transfer along the 200 Å hydrophilic axis. Moreover, it was suggested that the two “chemical” protons required for the quinone reduction are transferred through the so-called E-channel toward the quinone cavity. This “Nqo12-only” model was, in part, deduced from the higher local hydration of the terminal antiporter-subunit relative to the other antiporter subunits. The experimentally resolved water structure has a large variation between different Complex I isoforms and collectively indicates proton input sites in Nqo12, Nqo13, Nqo14, and Nqo8, together with a possible P-side output site reported for Nqo14.^35^ However, it is well-known that highly hydrated protein structures can also fully block proton transport, as in aquaporin,^36^ while transient hydration can lead to effective proton conduction across the membrane.^37^ Thus, the proton transfer activity cannot be deduced from the water structure alone but requires a detailed structural and energetic exploration of the hydration dynamics and proton transfer reactions.

Recently, Beghiah et al.^29^ isolated the individual antiporter modules from Complex I and showed that the dissected protein constructs contain all functional elements required to transport protons across proteoliposome membranes (Figure 1B,C). Specifically, both the ion-pair elements and titratable (Lys, His, and Glu) residues along the putative proton pathways are central for the proton transport process, consistent with the mechanistic requirements of the wave-propagation model. Similar direct evidence involving mutagenesis of central residues along the proton channel of the intact Complex I has been difficult to achieve based on experiments of the intact Complex I due to the long-range inhibition of the coupled proton and electron transport activity upon mutation of central residues along the proton channel.^9,10,19,21,38,39^

Generally, proton pumps employ molecular gates along the pathway that control the charge transfer reaction by opening and closing the proton conduits as a response to a redox or protonation “signal” to prevent the proton backflow along the thermodynamically favored direction. At a molecular level, such gates can be achieved by the interplay between water molecules and nonpolar residues that block the formation of Grotthuss-like proton arrays upon conformational changes. To address the molecular principles underlying the gating mechanism and *trans*-membrane proton transport in the antiporter modules of Complex I, we combine here directed mutagenesis and time-resolved spectroscopy, with proteoliposome experiments, *in vivo* assays, and atomistic molecular dynamics simulations.

## Results

### Identification of Gating Residues Based on Molecular Simulations

To probe the molecular principles underlying proton transfer in the antiporter modules, we performed atomistic molecular dynamics (MD) simulations of the Nqo12 and Nqo13 modules embedded in a lipid membrane (see Methods). Nqo12 was trimmed by removing the N-terminal amphipathic helix segment (residues 516–606), resulting in the modified Nqo12^ΔTM^ module (from here on referred to as Nqo12). In addition to the microsecond MD simulations of the WT constructs (see Methods for technical details), we also studied the effect of proton transfer-induced conformational and hydration changes by performing MD simulations, in which we stepwise shifted the proton along titratable residues within the established pathway, and as guided by previous quantum/classical (QM/MM) simulations^17,29,40^ (*cf*. also ref 41).

Both Nqo12 (Figure 2A) and Nqo13 (Figure 2D) establish transient S-shaped water wires across the membrane segment (see also Figures S1, S4, S7, S11 **)**, consistent with previous observations.^17,29^ As expected for a tightly gated proton pump, the water wires are not continuous but dynamically form and break and bridge the titratable conserved residues along the pathway (Figure 2B,E). The connectivity strongly depends on the protonation state of the titratable sites itself (Figures S1, S4, and S11), with the protonation state of the residue regulating the contact to the next site. We observe that the hydration dynamics correlate with the formation of an orientated intrinsic electric field along the pathway (Figures 2B,E and S2, S3, S5, S6, and S8), suggesting that the wetting transition is regulated by electrostatic effects (*cf*. also refs 42–49 ).

**Figure 2:**
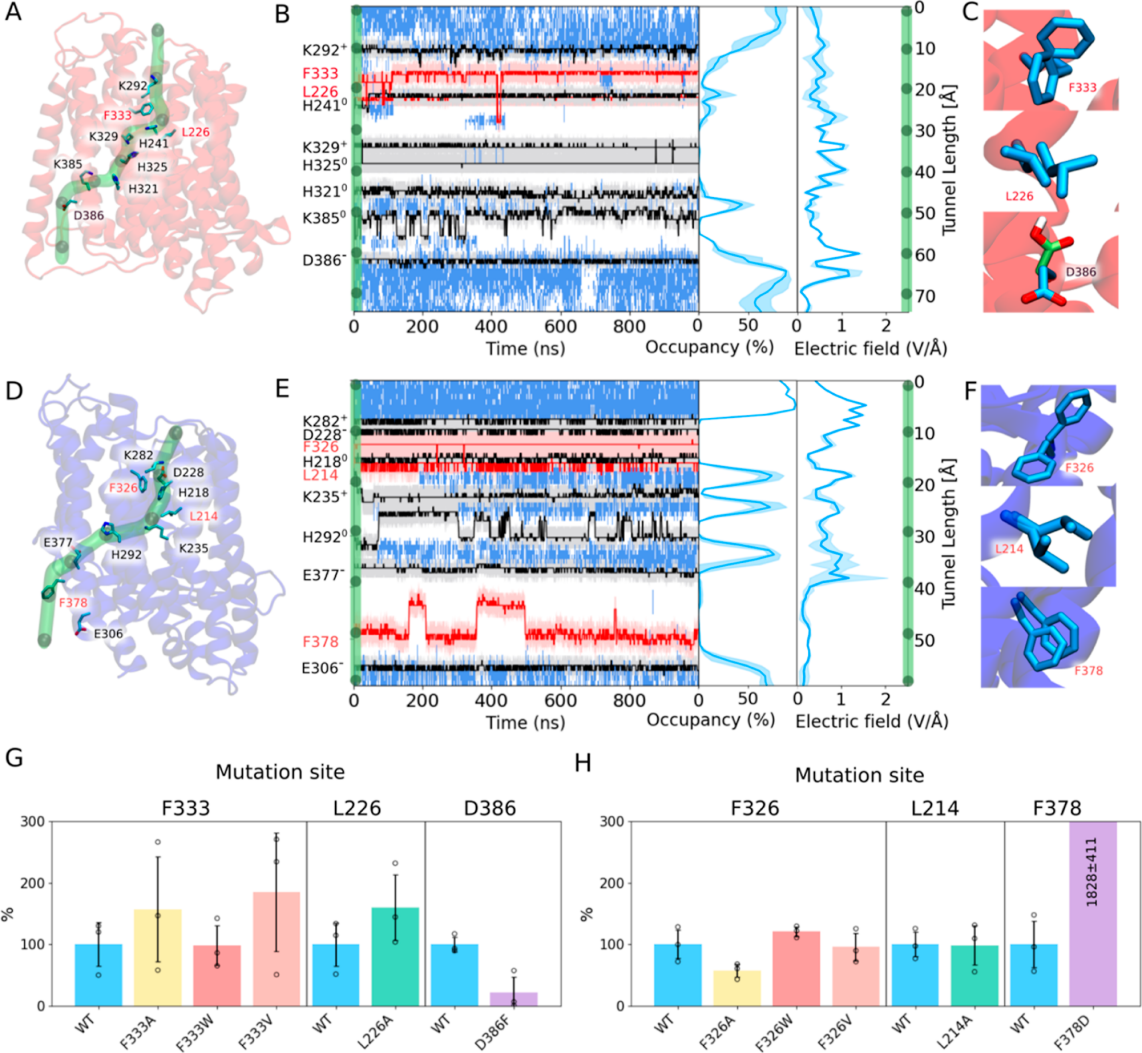
Hydration and electric fields along the proton pathway in Nqo12 and Nqo13 based on MD simulations. (A) Nqo12 and (D) Nqo13 with titratable residues labeled in black, gating residues in red, while the proton pathway is shown by a green tunnel. (B) Nqo12 and (E) Nqo13 shows hydration in the proton pathway, with the projected positions of the amino acid headgroups of conducting (black) and gating (red) residues, and their hydrodynamic radius shown as semitransparent gray/red. Middle: the occupancy of water molecules along the proton pathway (averaged over triplicate simulations). Right: Magnitude of the electric field strength along the channel (averaged over triplicate simulations). Snapshots showing different conformations of gating residues in (C) Nqo12 and (F) Nqo13. Normalized averaged water occupancy over three replicas with standard deviation at the site of mutation indicated above for (G) Nqo12 and (H) Nqo13 variants.

In Nqo12, the proton pathway forms via K292^12^ to H241^12^/K329^12^, with F333^12^ and L226^12^ regulating the hydration between the N-side input channel and the protein interior. The pathway continues via H325^12^ and H321^12^ to K385^12^, which connects via D386^12^ to the P-side bulk. The overall pathway is occupied by several water molecules, although we observe ca. 10 Å gaps between intermediate sites (at channel coordinates, *R* = 10–20 Å, 25–35 Å, and 50–58 Å, Figure 2B), whereas upon proton transfer along the pathway, we observe conformational changes of the central titratable residues (K292^12^, H241^12^, K329^12^, K385^12^, and D386^12^), bridging the hydration gaps (Figure S1). In particular, deprotonation of the central lysine residues, K329^12^ and K385^12^ (Figures S1D), which could occur upon proton transfer across the P-side exit site, leads to conformational fluctuations that favor the hydration of the N-side contacts. Moreover, nonpolar bulky residues along the transient hydration sites, in particular F333^12^ and L226^12^, transiently displace the water wires (Figures S9 and S10). This suggests that these nonpolar residues could establish local gating sites that regulate the proton transfer reactions (Figure 2A–F).

A qualitatively similar proton pathway also forms in Nqo13, bridging K282^13^/D228^13^ via H218^13^ to K235^13^, consistent with previous simulations,^10,14,17^ with nonpolar regions formed at F326^13^ and L214^13^ (*R* = 12 Å and 18 Å, Figure 2E). From the “central lysine residue”, K235^13^, the proton pathway continues via H292^13^ to E377^13^, which transiently connects to the P-side bulk by two possible output pathways (Figures 2D,E and S7). One of the pathways, located at an analogous position as the P-side exit in Nqo12, passes the dynamically flexible F378^13^, while another, minor pathway transiently forms a side-route via Y313^13^ to the P-side bulk. We note that dissecting Nqo13 from its neighboring subunits (Nqo12 and Nqo14) may introduce non-native lipid–protein contacts that could affect the local dynamics of the P-side output site, in particular along the latter side route (but see below). Interestingly, in some simulations, a Na^+^ ion, transferred from the P-side bulk, transiently interacts with E377^13^ (Figure S4G). However, this observation should not be taken as an indication that the antiporter is involved in sodium transport, as MD simulations employ fixed protonation states, and thus, the transient coordination of Na^+^ ion by E377^13^ likely indicates an underlying thermodynamic propensity for the residue to undergo a protonation change. In this regard, previous experiments show that the antiporter modules do not conduct Na^+^ ions across proteoliposome membranes.^29^

To test the functional role of the identified putative gating sites, we next replaced the residues in silico with either smaller (Ala and Val) or larger (Trp) nonpolar amino acids, more specifically, L226A^12^, F333A/V/W^12^, L214A^13^, and F326A/V/W^13^ (Figures 2G,H and S9–S12). Moreover, we probed the effect of substituting the terminal carboxylate with a phenylalanine (D386F^12^) in Nqo12, and *vice versa*, replacing the analogous phenylalanine with a carboxylate in Nqo13 (F378D^12^). We further compared these models with simulations mimicking a putative “open/conductive” channel conformation, established by the respective E132Q^12^ and E123Q^13^ substitutions, in which the “ion-pair opening” enhances proton transport along the central channel (*cf*. ref 29). As expected, the substitution of the bulky residues with smaller or polar amino acids (F333A^12^, F333V^12^, L226A^12^, L214A^13^, and F378D^13^) results in a local increase of the hydration along the pathway together with altered electric field components, resembling the *open* states in E132Q^12^ and E123Q^12^ (Figures 2G,H and S9–S12). For the F326A/V^13^ variants, we observe a subtle rearrangement of adjacent hydrophobic residues, where L385^12^ shifts to a position occupied by F326^13^ in the WT (Figures S10C,D and S12D **)**, filling empty gaps along the pathway and qualitatively preventing excessive hydration. Following similar principles, introduction of bulkier residues (D386F^12^ and F326W^13^) locally decreases the hydration level of the pathway, further supporting that the potential gating function of the identified residues (Figures S9G, S10E, and S11).

In addition to the structural rearrangement in the F326A^13^ and F326V^13^ variants, we observe a subtle shift in TM7a/b and H241^12^ in the F333A^12^ variant (Figures S9C, S10C,D, and S12D,G). This suggests that F333^12^ not only controls the hydration level of the pathway but could also affect the opening/closing-dynamics of the broken helix elements. Notably, H241^12^ shows an increased flexibility in several variants (F333A^12^, F333W^12^, and F333V^12^, Figures S9 and S12G), resembling the dynamics of the conductive state of the E132Q^12^ variant. In contrast, the F333W^12^ substitution seals the N-side input channel by the indole ring adapting a horizontal conformation along the channel (Figures 2G and S9E and S12H).

Of particular functional interest is also the swap of the terminal carboxylate/phenylalanine in Nqo12/13. In this regard, F378D^13^ shows a drastic increase in the hydration and electric field strength along the P-side pathway (Figures 2H and S10G). However, the resulting “overflooding” of the tightly controlled P-side exit pathway is significantly more drastic than that observed in the WT-Nqo12 model (Figure S9A), indicating that the F378D^13^ substitution could result in significant proton leaks (see below). Similarly, the D386F^12^ substitution results in a lower hydration level at the P-side output channel (Figures 2G and S9G), which correlates with a decreased electric field strength along the pathway, despite transient proton wires still being observed during the simulation.

Taken together, our MD simulations suggest that both Nqo12 and Nqo13 establish transient proton pathways across the membrane with contiguous hydrogen-bonded contacts between titratable residues, tightly controlled by the protonation state of the residues themselves. The protonation shifts induce local electric fields along the pathway (Figures S2, S3, S5, S6), controlling the hydration dynamics, and structurally supported by the identified bulky nonpolar residues. Our MD simulations identify putative gating sites along the input/output channels that regulate the formation of proton pathways, while their substitutions result in perturbed hydration states along the transient proton conduits.

### Bulky Gating Residues Modulate the Proton Transport Process across the Membrane

To experimentally assess the molecular principles underlying the proton gating process and test the effect of the computationally identified gating sites, we expressed and isolated the individual antiporter modules Nqo12 and Nqo13 and introduced mutations along these sites. The proton conduction was probed in proteoliposomes by co-reconstituting the individual antiporter modules together with *E. coli* ATP synthase, creating a proton gradient upon addition of ATP (Figure 3A). We applied here the recent lauryl maltose neopentyl glycol (LMNG) autoinsertion reintegration (LAiR) approach,^50^ which allows the establishment of tight proteoliposomes relying only on the use of the soft detergent LMNG, without the addition of cholate, which destabilizes the liposome membranes during the reconstitution. We monitored here the proton conduction using the pH-sensitive fluorescent dye pyranine (8-hydroxypyrene-1,3,6-trisulfonicacid, HPTS),^51^ encapsulated within the proteoliposomes—a ratiometric dye, in which the optical changes can be directly related to a pH shift, as established by calibration (Figure S13A). The proton transfer reaction was initiated by the addition of 0.04 mM ATP, which is hydrolyzed by ATP synthase, and generates a *pmf* by the ATP-driven proton pumping activity that acidifies the inner volume of the proteoliposomes, resulting in quenching of the pyranine fluorescence signal (Figure 3B,C), followed by dissipation of the generated *pmf* by the addition of the protonophore gramicidin (Figure 3B,C).

**Figure 3:**
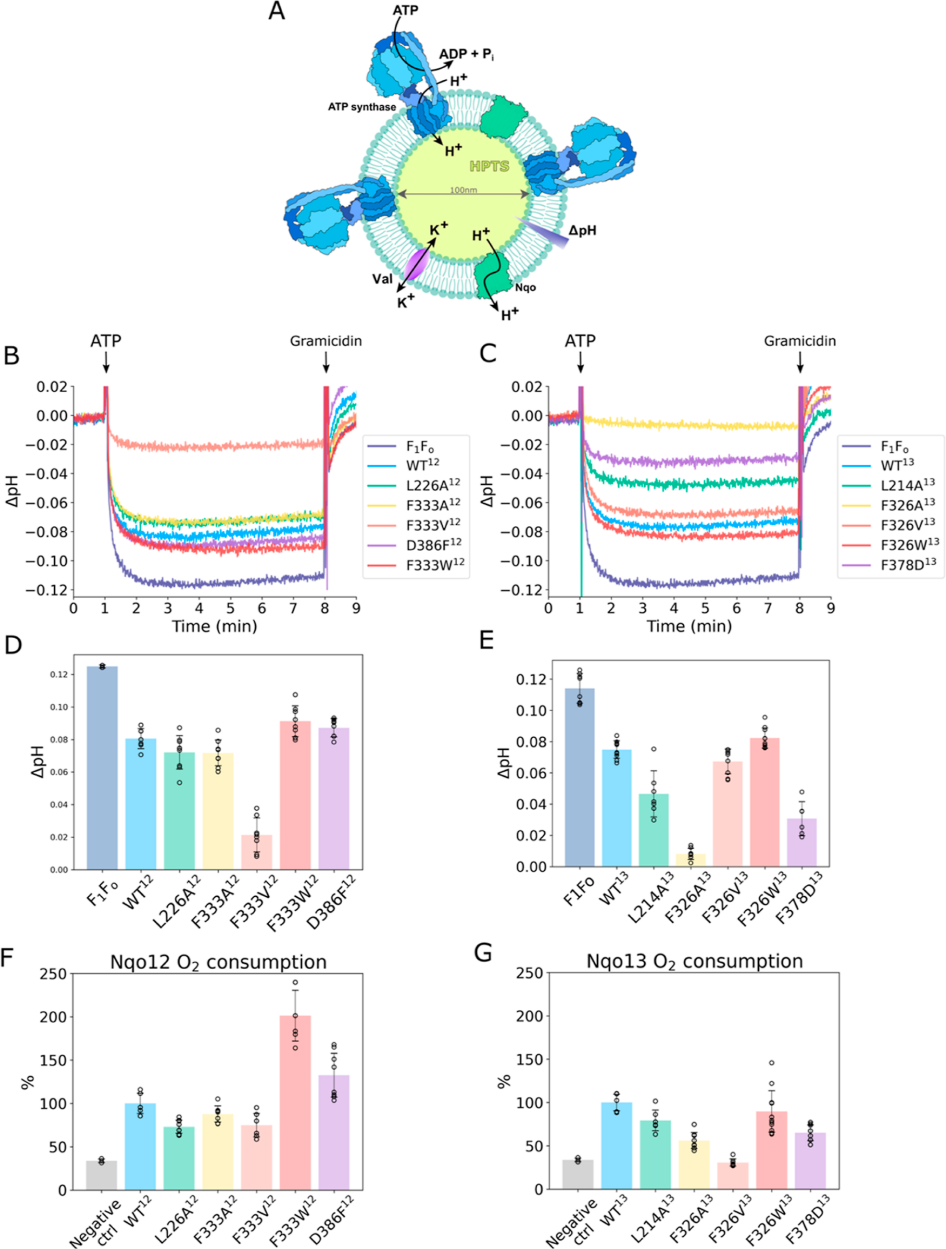
Proton conduction assays, monitoring ΔpH in proteoliposomes using pyranine and O_2_ consumption in isolated membranes. (A) Proteoliposomes setup: 100 nm proteoliposomes with incapsulated pyranine (HPTS) were reconstituted with ATP synthase and antiporter modules in a molar ratio of 3:2, and valinomycin added prior to measurements. (B,C) Proton conduction measurements for (B) Nqo12 and (C) Nqo13, following pyranine fluorescence. Addition of ATP results in the generation of a proton motive force by ATP synthase, which drives the proton transport though the antiporter module, and results in a decrease of pyranine fluorescence, which was converted into ΔpH via pH-calibration (Figure S13). Gramicidin was added at the end of the experiment to dissipate the generated proton gradient. (D,E) Bar plots of established ΔpH for (D) Nqo12 and (E) Nqo13 based on exponential fits of the proton transport data (see Methods, Figure S18). (F,G) Oxygen consumption of antiporter modules in isolated membranes for (F) Nqo12 and (G) Nqo13, quantified using an oxygen electrode. NADH was added to start the reaction, and the O_2_ consumption rate was calculated from a linear fit (see Methods, Figure S18). The oxygen consumption was normalized for the total protein concentration and the available NADH oxidizing dehydrogenases (see Methods). The negative control refers to the O_2_ consumption in isolated *E. coli* membranes without expression of antiporter constructs.

To study the proton conduction of the antiporter variants, we co-reconstituted them into the ATP synthase-proteoliposomes. We observe that Nqo12 and Nqo13 constructs kinetically compete with the proton pumping activity of ATP synthase, with the proton conduction resulting in a smaller ΔpH, with Nqo12^WT^ (ΔpH = 0.081) and Nqo13^WT^ (ΔpH = 0.075) (Figure 3B–E), supporting our previous experiments conducted using the semiquantitative 9-amino-6-chloro-2-methoxyacridine (ACMA) dye.^29^

Mutation of the identified nonpolar gating residues along the proton pathways into smaller or bulkier hydrophobic residues strongly modulates the rate of proton conduction in the antiporter modules. In this regard, upon introduction of a smaller side chain along the putative gating sites, we observe a significantly faster proton conduction rate and a smaller ΔpH relative to the WT antiporter module in the L226A^12^ (ΔpH = 0.072), L214A^13^ (ΔpH = 0.046), F333A/V^12^ (ΔpH = 0.072/0.021), and F326A/V^13^ (ΔpH = 0.008/0.067) variants (Figure 3D,E). Interestingly, in the F333V^12^ and F326A^13^ variants, the conduction rate is notably faster relative to the other variants, especially F333A^12^ and F326V^13^, suggesting that these substitutions result in an impaired gating function. In contrast, introduction of a bulkier side chain at the gating site results in a reduced proton conduction rate (i.e., larger ΔpH) relative to the WT modules in F333W^12^ (ΔpH = 0.090) and F326W^13^ (ΔpH = 0.082). Moreover, as expected from our simulations, the D386F^12^ substitution resulted in a slower proton conduction rate relative to the WT module (ΔpH = 0.087), while the F378D^13^ substitution shows enhanced proton conduction (ΔpH = 0.031) (Figure 3D,E).

### Probing the Kinetics of Proton Transport by Stopped-Flow Spectroscopy in Proteoliposomes

We next applied stopped-flow spectroscopy to resolve the millisecond kinetics of the proton transport reaction in the Nqo12 and Nqo13 variants (Figure 4A). To this end, we followed the generation of the membrane potential upon addition 1 mM ATP with ATP synthase/Nqo-proteoliposomes, monitoring absorption changes of oxonol VI,^52^ with a time-resolution of 2 ms, established within the stopped-flow apparatus. The absorbance change of oxonol VI correlates with the buildup of a *trans*-membrane electrical potential (Figure S13B), established by the ATP synthase. We obtain an initial rate of Δψ formation of ∼120 mV s^–1^ with ATP synthase alone, which is comparable to the transfer of ∼210 H^+^ s^–1^, as estimated based on the size and capacitance of the liposomes (see Methods). Upon co-reconstitution of the WT antiporter modules with ATP synthase, we obtain a significant reduction of the electrical gradient across the membrane, resulting in rates of 53.5 mV s^–1^ and 26.7 mV s^–1^ for Nqo12 and Nqo13, respectively (Figure 4B–E). Substitution of the gating residues to smaller hydrophobic side chains or to protonatable residues results in an a smaller buildup of Δψ, consistent with an increased conduction rate of L226A^12^ (45.0 mV s^–1^), F333A/V^12^ (40.7 mV s^–1^/9.95 mV s^–1^), L214A^13^ (14.0 mV s^–1^), F326A/V^13^ (15.4 mV s^–1^/10.8 mV s^–1^), and F378D^13^ (22.1 mV s^–1^), whereas substitutions to bulkier residues result in a similar or increased buildup of the Δψ in F333W^12^ (51.3 mV s^–1^) and F326W^13^ (28.4 mV s^–1^), while the D386F^12^ variant has a slower initial rate of 43.8 mV s^–1^ relative to the WT, but an overall similar Δψ as the WT (Figure 4B–E). Taken together, our stopped-flow experiments show that the antiporter modules Nqo12 and Nqo13 kinetically compete with transport of protons by ATP synthase and that the bulky gating residues control the rate of *pmf* formation across the proteoliposome membranes, supporting the molecular observations derived from our MD simulations.

**Figure 4:**
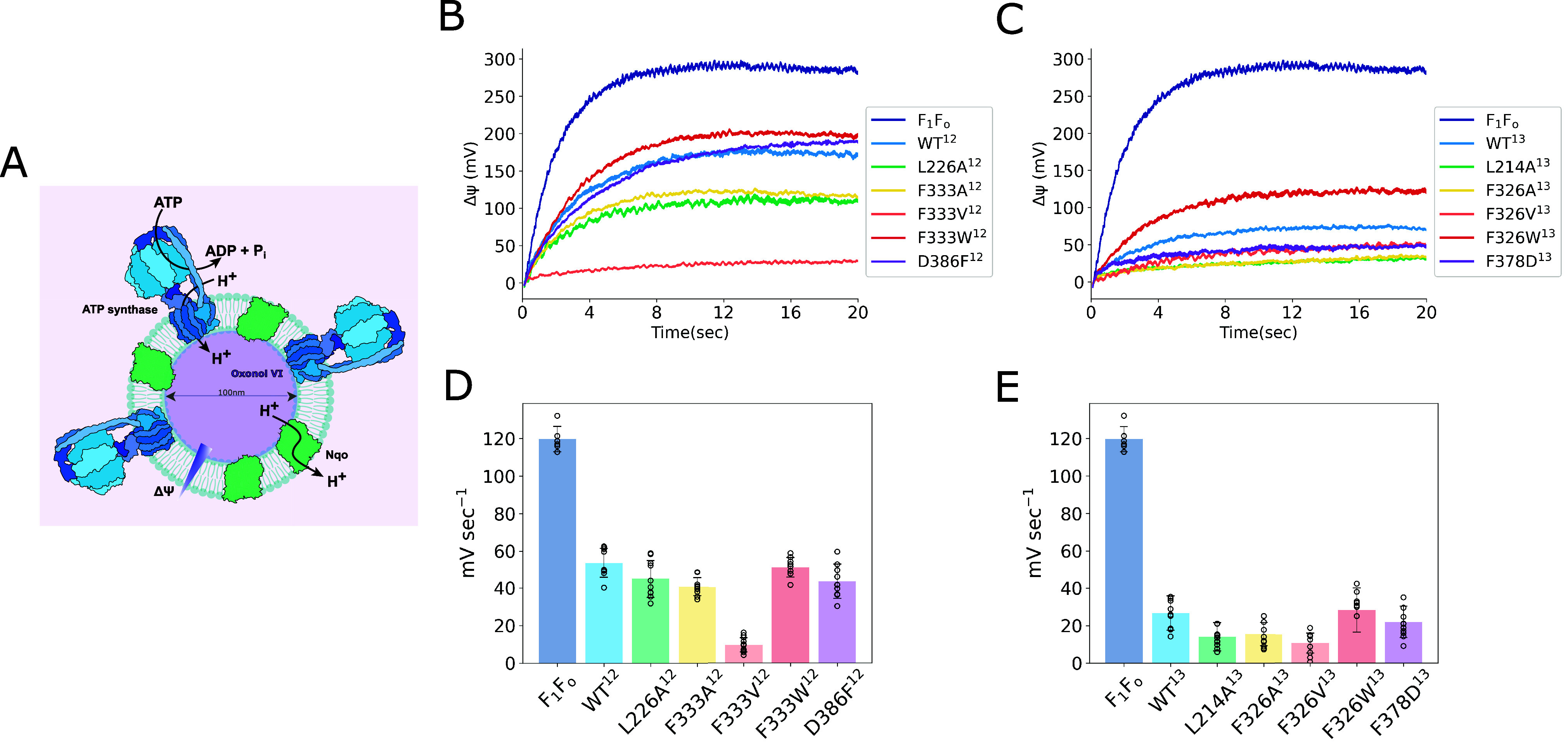
Proteoliposomes assay, monitoring Δψ with stopped-flow spectroscopy and oxonol VI. (A) Proteoliposomes setup: 100 nm proteoliposomes, resuspended in buffer with oxonol VI, were reconstituted with ATP synthase and antiporter modules in a molar ratio of 3:4. Proton conduction measurements for (B) Nqo12 and (C) Nqo13, following oxonol VI absorbance difference at 588 and 625 nm. Addition of ATP resulted in the generation of a proton motive force by ATP synthase, which drives the proton transport through the antiporter module and results in a decrease in oxonol VI absorbance, which was converted into Δψ buildup (in mV) based on calibration (Figure S13B). Bar plots of established initial rates (mV s^–1^) of Δψ buildup for (D) Nqo12 and (E) Nqo13, obtained by linear fits (see Methods, Figure S18).

### Probing the Proton Conduction in Isolated Membranes

To test the possible proton conduction properties of the antiporter modules in respiring membranes, we next assessed the rate of respiration in isolated *E. coli* membranes upon overexpression of the antiporter modules. In this regard, we measured the oxygen consumption level during cellular respiration using a Clark oxygen electrode upon addition of NADH as an electron donor, while quantifying the expression level of the antiporter modules by the fluorescence of sfGFP, attached to the modules (see Methods). The expression of the proton conducting antiporter modules in the respiring membranes is expected to impair the formation of a *pmf* and thus to be harmful for the energy metabolism of the cell. The bacteria compensate by lowering the expression level of the antiporter modules, as monitored here by the GFP fluorescence (Figure S15). Overall, we find that the fast proton-conducting modules with decreased gate regions result in a decreased respiration and lowered expression level of the antiporter module (Figure S15), while upon expression of a slower proton conducting variant with the increased gate region, we observe a higher respiratory rate, enabling higher expression levels of the variant (Figure S15), with the exception of F326W^13^ (see below). The faster proton conducting modules correlate with a decrease in the rate of oxygen consumption relative to the WT construct (100%), for L226A^12^ (73 ± 7%), F333A/V^12^ (88 ± 9%/75 ± 13%), L214A^13^ (79 ± 12%), F326A/V^13^ (56 ± 9%/31 ± 4%), and F378D^13^ (65 ± 9%) (Figure 3F,G), while for the slower conducting modules, we observe an overall increase in the rate of oxygen consumption relative to the WT (100%) for F333W^12^ (201 ± 29%) and D386F^12^ (132 ± 25%), with the exception of F326W^13^ (91 ± 24%) (Figure 3F,G), where the substitution could result in a less stable protein construct. Taken together, these findings support that the antiporter modules conduct protons also in respiring membranes.

## Discussion

Here, we have addressed mechanistic principles of proton transport in dissected antiporter modules of Complex I by combining atomistic MD simulations with site-directed mutagenesis experiments, spectroscopic studies in proteoliposomes, and assays of respiration in isolated membranes. On a general level, our findings highlight the role of tightly regulated water wires that transiently connect titratable residues and provide stepwise proton conduits across the membrane (Figure 5A). We find that the hydration process is mediated by local electric field effects, and it is structurally supported by nonpolar gating residues that allow for controlled wetting/dewetting transitions (Figure 5A,B). These observations may also rationalize why several high-resolution cryo-electron microscopy (cryo-EM) as well as large-scale MD simulations reveal only partial “protonic” connectivity across the membrane domain of Complex I. Indeed, such tightly controlled kinetic gates are central for the function of several redox-active proton pumps^24,44,47,53,54^ (Figure 5C), with continuous proton conduits resulting in proton leaks.

**Figure 5:**
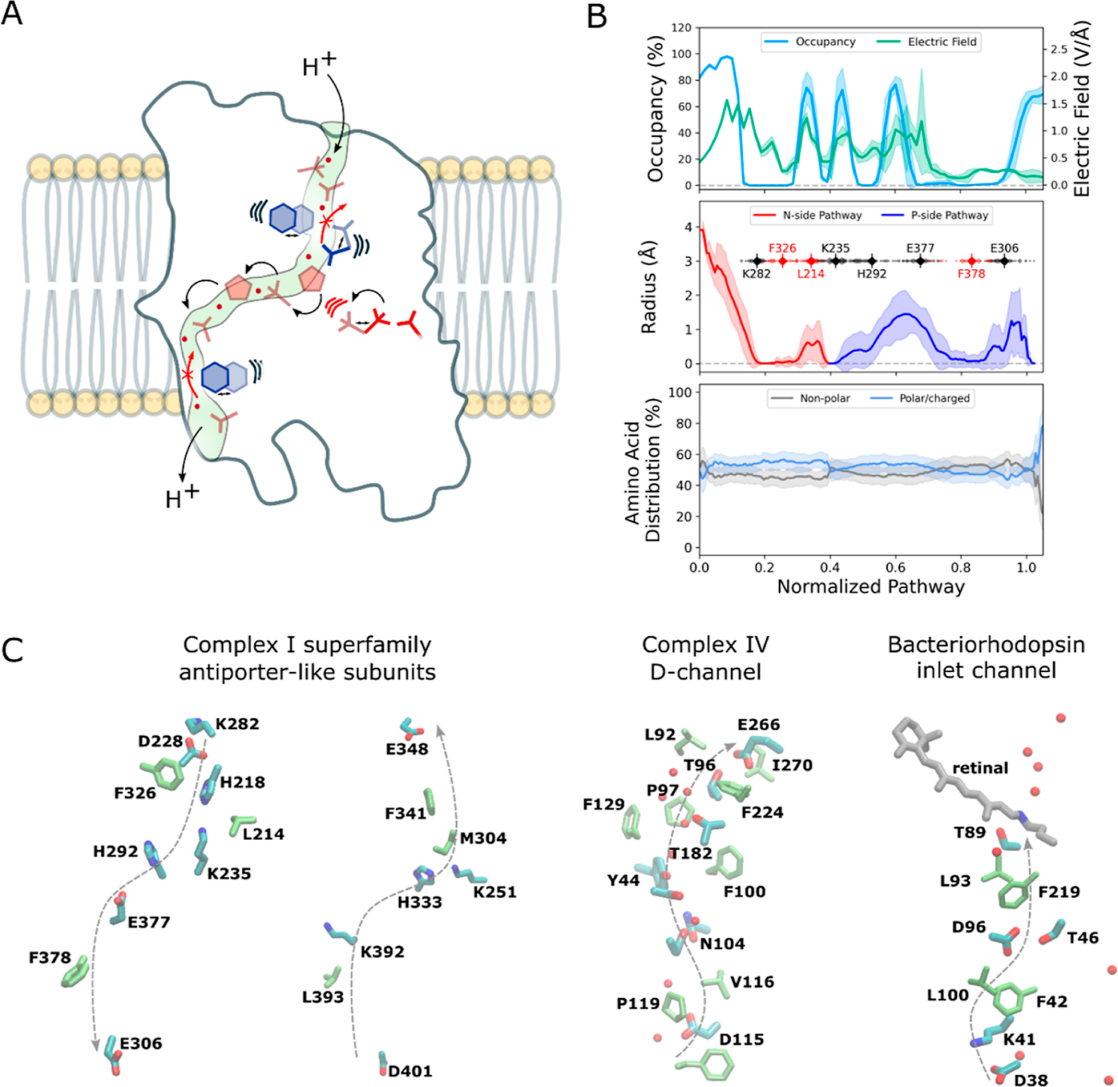
Schematic model of proton gating in antiporter-like subunits of Complex I. (A) Water-mediated proton transport takes place via titratable residues and water molecules (in red), as a response to opening of the conserved ion pair. The central nonpolar bulky residues (in blue) regulate proton transfer along the pathways, by creating local gating sites that prevent leak reactions. (B) *Top*: Water occupancy and electric fields along the proton pathway; *Middle*: Channel radius along the pathway (excluding the titratable residues themselves) in Nqo13; *Bottom*: Architecture of the proton channel, with distribution of nonpolar and polar residues along the pathway, based on statistical analysis antiporter-like subunits from various organisms (see Methods). (C) Structure of proton pathway in antiporter-like subunits of Nqo13 (PDB ID: 4HEA) and MrpA complex (PDB ID: 7D3U); the proton-conducting D-channel of cytochrome *c* oxidase (PDB ID: 8OVD/8OVC), and proton inlet channel in bacteriorhodopsin (PDB ID: 5B6V). The figure shows both titratable residues and putative nonpolar gating residues.

We further probed the role of nonpolar gating residues, identified based on MD simulations and studied in our biophysical experiments. Our simulations suggested that the hydration process is directed by the charge transfer process itself, with the introduction of mutations resulting in perturbation of the local proton gates, guided by simulations and shown in our mutagenesis work. On a general level, these findings could provide a basis for the exploration of how the protein and hydration dynamics are linked to the free energy landscape of proton transfer itself (*cf*. also ref 40). Experimentally, we achieved highly controlled conditions to study proton transport in the dissected antiporter modules by adapting the recent LMNG-based reintegration autoinsertion method,^50^ which allowed us to create proteoliposomes that can sustain large (*ca*. 200 mV) proton gradients, enabled by the spectroscopic quantification with pyranine and oxonol VI to monitor the charge transport up to millisecond time scales. This proton conduction likely arise from a weighted averages over proton transfer in two directions, as the antiporter constructs have a preferred orientation, with the N-side channel facing *ca*. 75% the interior of the proteoliposomes.^29^ While the antiporters transport protons in both directions in the intact Complex I, it remains currently unknown if the conduction rates are symmetrical in both directions. In this regard, it could be possible to experimentally control the orientation of the antiporter modules by the lipid composition and/or charge, as shown, *e.g.*, for *bo*_3_ oxidase,^55^ and thus probe differences in the forward and backward conduction rates.

Our experiments further support that the proton transport is controlled by the formation of transient water wires. Introduction of smaller residues (Phe to Val/Ala) resulted in significantly enhanced proton conduction rates, while larger residues (Phe to Trp) lowered the overall proton conduction. These findings are also consistent with previous work,^14,21^ suggesting that the redox-coupled proton-pumping activity of Complex I is affected by substitution of residues at the entrance of the N-side proton channels. While all antiporter variants resulted in stable protein constructs (Figure S14), our MD simulations suggest that certain mutations may introduce subtle conformational changes in the structure (Figure S10). Our MD simulations also suggested that the proton transfer along the channel as well as mutation of gating residues can introduce wetting/dewetting transitions up to 20–30 Å away from the site of mutation, indicating that allosteric effects could be involved in the proton transfer process.

Our simulations suggest that the μs-protein dynamics of the putative gating sites is controlled by intrinsic protonation states of central titratable residues along the pathway. These intrinsic protonation states could thus locally favor the gate opening and shift the free energy of the wetting transition, which in turn regulates key protonation steps that determine the overall rate of proton transport across the membrane on millisecond time scales. More generally, these findings reflect that the protein function is connected through a hierarchy of different time scales, from the fast nano/microsecond time domain to slower milliseconds, as also observed in enzyme catalysis.^56^

Interestingly, similar nonpolar constriction sites, as described here, are also found in other systems, such as in the related multiresistance and pH-adaptation (Mrp) antiporters, but also in Complex IV (cytochrome *c* oxidase), and bacteriorhodopsin (Figure 5C). In addition to a steric hindrance linked to the hydration transitions, such residues could also provide a unique dielectric environment, supporting the proton transfer reactions. The role of transient water wires has also been supported in *de novo*-designed minimal proton channels,^37^ suggesting that our findings could guide the future design of synthetic proton pumps.

Based on direct experimental evidence, it has remained difficult to establish which antiporter modules are directly involved in transporting protons, as mutagenesis affects the global proton-coupled electron transfer (PCET) reactions and results in an impediment of the global PCET-machinery upon substitution. Here, we have circumvented this challenge by studying the dissected antiporter modules based on a theory-guided bottom-up approach. Our findings provide mechanistic details underlying the elusive long-range proton pumping function of Complex I.

Of particular mechanistic interest is the terminal Nqo12 subunit presenting an additional helix (TM15) that together with a terminal D386^12^ could favor the release of the proton and result in initiation of the protonation backwave (cf. ref 23). Indeed, early MD simulations^57^ found a significant hydration of the P-side exit channel in Nqo12, while simulations of the entire Complex I^14^ revealed also ensembled averaged water positions in other antiporter subunits. Based on the higher hydration of Nqo12 relative to the other antiporter subunits observed in these simulations,^14,58^ Zhang and Li^51^ suggested that only the terminal Nqo12 subunit could be involved in the proton transport. Indeed, Sazanov and co-workers^7^ adopted a similar argument based on the “Nqo12-only” (NuoL/ND5-only) proton pumping model, where four water molecules are experimentally resolved between D386^12^ and the P-side bulk. Moreover, Parey et al.^15^ compared cryo-EM data and MD simulations and suggested a variation of a “Nqo12-only” like pumping model, with two protons collectively taken up by the three antiporter-subunits, but also transported along the terminal Nqo12 (*cf*. also ref 59 for a revised proposal).

Our current findings challenge the “Nqo12-only” model, as we can quantify a tightly regulated proton transport activity across proteoliposome membranes for both Nqo12 and Nqo13 that is strongly affected by mutation at the identified gating sites. Our findings are thus also in line with removal of ND5 (Nqo12) and ND4 (Nqo13) from the intact Complex I that results in approximately half of the proton pumping activity^11^ and generally support that all antiporter subunits (Nqo12/NuoL, Nqo13/NuoM, Nqo14/NuoN, and Nqo7/8/10/11/1/NuoA/J/K/H) catalyze proton transport *across* the membrane. Moreover, our findings show that introduction of a charged terminal residue in Nqo13 resulted in an uncontrolled water entry along the P-side channel (Figures 2H and S10G) and a significant increase in the proton conduction rate (Figure 3C,E), which is expected to lead to an uncontrolled redox-driven proton pumping activity for the full Complex I. In Nqo12, we suggest that the terminal charged residue is shielded by the additional helix, with a potential function in the reversal of the proton pumping machinery, *viz.,* during the proposed backwave. Moreover, the substitution of the terminal charged residue in Nqo12 with a phenylalanine residue resulted in lowered hydration at the P-side channel (Figures 2G and S9G), and decreased, but not depleted proton conduction rate (Figure 3B,D), thus reinforcing the notion that the same terminal residue in Nqo13 controls proton conduction and does not arise from dissection of the construct from the full Complex.

The dissected antiporter modules also conduct protons in native membranes which has interesting consequences during the assembly of the membrane domain of Complex I. As shown here, the expression of the dissected modules results in an uncontrolled respiratory rate that correlates with the rate of proton conduction in the antiporter constructs (Figure 3F,G). During assembly of the complete Complex I machinery, we speculate that the antiporter subunits could be capped by assembly factors that prevent them from dissipating the *pmf*. Indeed, putative assembly factors, such as NDUFAF1 and CIA84 that bind to ND2 and NDUFC2 (in yeast) together with the cardiolipin remodeling enzyme Taffazin,^60^ could be involved in providing such “lids” for the proton channels.

Taken together, our combined findings highlight the intricate interplay between transient hydration of proton channels and kinetic gates in controlling *trans*-membrane proton transfer across the antiporter modules of Complex I.

## Conclusions

By combining stopped-flow spectroscopy and mutagenesis experiments with MD simulations, we showed here that the terminal (Nqo12/NuoL/ND5) and penultimate (Nqo13/NuoM/ND4) antiporter subunits conduct protons across membranes by forming transient proton wires connecting titratable conserved residues. We identified nonpolar gating residues at constriction sites, which provide central regulation sites, and could establish a basis for the tightly controlled long-range energy transduction mechanism in the intact Complex I. Moreover, we find that a charge swap between terminal residues in Nqo12 and Nqo13 support that both antiporter constructs conduct protons, while also showing intrinsic differences in their conduction properties. On a general level, our theory-guided experiments reveal mechanistic principles underlying electric field effects and hydration dynamics in controlling long-range charge transport in biological systems.

## Materials and Methods

### Expression and Purification of Antiporter Constructs

The antiporter constructs, Nqo12 and Nqo13, were expressed and purified following our previously developed protocol (see ref 29). In this regard, the pWALDO^29,61^ -Nqo constructs were transformed and overexpressed into the Lemo21,^62^ *E. coli* competent cells. TB media supplemented with l-rhamnose (0.1 mM for Nqo12 and 0.5 mM for Nqo13), chloramphenicol (30 μg mL^–1^), and ampicillin (100 μg mL^–1^) were prepared, and cultures were left at 37 °C until induction with IPTG (0.4 mM) at OD_600_ = 0.5, following a temperature decrease to 25 °C, and culture grown overnight. Cells were harvested and homogenized in a resuspension buffer (25 mM HEPES pH 7.5, 150 mM NaCl). The lysis was performed using an Emulsiflex, and cell debris was removed by high-speed centrifugation, 25,000 *g* for 20 min at 4 °C. Membranes were collected by ultracentrifugation, at 250,000 *g* for 2 h at 4 °C. Membranes were solubilized in 2% LMNG at 6 mg mL^–1^ total protein concentration and left to stir for 1 h before removing insolubilized membranes by ultracentrifugation at 250,000 *g* for 30 min at 4 °C. The supernatant was loaded several times into a 5 mL His-Trap HP column (Cytiva), pre-equilibrated with purification buffer (25 mM HEPES pH 7.5, 300 mM NaCl, 20 mM imidazole). The column was washed extensively with different imidazole concentrations (20, 70, 125, and 150 mM), and the elution was performed with 300 mM imidazole. The purified protein was loaded into a HiLoad 16/600 Superdex 200pg for size-exclusion chromatography. Relevant fractions were pooled and concentrated, with the protein concentration measured using a NanoDrop spectrophotometer.

### Design of the Gating Mutations

Single-point mutations were introduced using designed primers (Thermo Fisher) (Table S1) into the pWALDO-Nqo expression plasmid and confirmed by DNA sequencing (Eurofins, Uppsala, Sweden).

### Expression and Purification of *E. coli* ATP Synthase

ATP synthase was purified using a protocol similar to those established before (cf. ref 29). Briefly, expression of the pFV2 plasmid was performed in DK8 *E. coli* competent cells. Cells were grown in LB medium supplemented with ampicillin (100 μg mL^–1^) and MgCl_2_ (1 mM) overnight. Harvested cells were resuspended in lysis buffer (50 mM HEPES pH 8.0, 100 mM NaCl, 5% glycerol). Lysis was performed using an Emulsiflex, and cell debris was removed by high-speed centrifugation, at 25,000 *g* for 20 min at 4 °C. Membranes were collected by ultracentrifugation at 250,000 *g* for 1.5 h at 4 °C. The membranes were solubilized using 2% LMNG in resuspension buffer (50 mM HEPES pH 7.5, 200 mM KCl, 150 mM sucrose, 0.8% Type II–S soybean lipids) at a concentration of 1.5 mL per grams of cells, before removing insolubilized membranes by ultracentrifugation (250,000 *g*, 30 min, 4 °C). The supernatant was loaded twice into a 5 mL His-Trap HP column (Cytiva) and pre-equilibrated with the purification buffer (50 mM HEPES pH 7.5, 200 mM KCl, 150 mM sucrose, 0.005% LMNG, 20 mM imidazole). The column was washed in two steps with the purification buffer supplemented with 20 and 90 mM imidazole before elution using 285 mM imidazole. Relevant fractions were pooled and concentrated. The purified protein was loaded into a HiLoad 16/600 Superose 6pg (Cytiva) for size-exclusion chromatography. Relevant fractions were pooled and concentrated. Concentration was measured by using a NanoDrop spectrophotometer.

### Protein Reconstitution into Proteoliposomes with Pyranine (HPTS)

Coreconstitution of purified ATP synthase and antiporter constructs was performed by preparing liposomes with soybean Type II–S lipids, resuspended in proteoliposome buffer (2 mM HEPES at pH 7.3, 50 mM KCl, 5 mM MgCl_2_) at 5 mg mL^–1^. Resuspended lipids were freeze–thawed 7 times to disrupt multilamellar lipid formations. Pyranine (HPTS—8-hydroxypyrene-1,3,6-trisulfonic acid trisodium salt) was incorporated into the liposomes by freeze–thawing 3 times the lipids, with 2 mM HPTS final concentration. The liposome size was selected by extruding the lipids 21 times against a membrane of 100 nm pore diameter (Nuclepore membranes, Whatman Ltd.). ATP synthase and the Nqo constructs (in a molar ratio of 3 ATP synthase to 2 Nqo subunits, 200:1 (w/w) lipid to protein) were added to 0.5 mg of preformed liposomes and inserted following the LMNG autoinsertion reintegration (LAiR) approach.^50^ Reconstitution was left at room temperature for 40 min before removing external pyranine using a PD-10 column (Cytiva). Proteoliposomes were pelleted down by ultracentrifugation for 30 min, at 50,000 rpm, 4 °C, and resuspended with proteoliposomes buffer at a lipid concentration of 20 mg mL^–1^.

### Proton Conduction Measurements Using Pyranine (HPTS)

Proton conduction for the antiporter constructs was followed by pyranine fluorescence using a Cary Eclipse Fluorescence Spectrophotometer (Agilent Technologies). Excitation wavelengths for HPTS were set at 405 and 460 nm, while emission was followed at 510 nm. The proton conduction was assayed in plastic cuvettes (*d* = 1 cm) using 5 μL of proteoliposome resuspension, supplemented with 1 nM valinomycin to a final volume of 500 μL in the proteoliposomes buffer. The baseline was equilibrated at 37 °C until a stable signal was achieved. The assay was started by addition of 0.04 mM ATP after the first minute and ended by addition of gramicidin after 7 min to dissipate the generated ΔpH. The proton conduction was estimated from the ratio (*R*) of HPTS emission at 460 and 405 nm, *R = I* ^460^ */I* ^405^, and related to its pH value based on

$$pH=a\cdot \log_{10}\left(R\right)+b$$

where parameters *a* and *b* were obtained from the calibration curve (Figure S13), yielding *a* = 1.931 and *b* = 7.926. The ΔpH values were calculated as the difference between the initial pH, before addition of ATP, and final pH, before dissipation with gramicidin. The initial pH was taken as the baseline average over the first minute, and the final pH was obtained by fitting the assay curve with a single exponential function. The data were measured from *n* = 6–10 independent measurements. The time resolution of the HPTS experiments was 600 ms.

### Stopped-Flow Experiments Using Oxonol VI

Proteoliposomes were prepared from lipid resuspension (5 mg mL^–1^) by 7 freeze–thaw cycles before extrusion using a membrane with a pore diameter of 100 nm (Nuclepore membranes, Whatman Ltd.). The proteins were reconstituted using the LAiR method^50^ with an ATP synthase-to-antiporter molar ratio of 3:4. The reconstituted system was left at room temperature for 40 min before proteoliposomes were pelleted down by ultracentrifugation for 30 min, 50,000 rpm, at 4 °C. The proteoliposomes were resuspended in the proteoliposome buffer, supplemented with 5 μM oxonol VI (Sigma-Aldrich) at a lipid concentration of 0.2 mg mL^–1^. The proteoliposomes were loaded on a stopped-flow apparatus (*Applied Photophysics*), together with 2 mM ATP, resuspended in the proteoliposome buffer with oxonol VI, and resulting in a final ATP and proteoliposome concentration of 1 mM and 0.1 mg mL^–1^, respectively, in the mixing cuvette. The absorption signal of oxonol VI was monitored for 20 s at 588 and 625 nm, and 10–15 measurements were averaged per sample, to relate to the buildup of Δψ across the proteoliposome membrane, based on the calibration (Figure S13). The calibration was performed by relating Δ*A*_588nm–625nm_ to Δψ, with known Δψ, established by different potassium concentrations across the liposome membrane (Figure S13, Table S4). Initial rates were obtained by using a linear fit over the first second of the reaction. The time-resolution of the stopped-flow experiments was 2 ms.

### Oxygen Consumption Measurements in Membranes and sfGFP Scan

Oxygen consumption of isolated membranes comprising the expressed antiporter constructs was measured with a Clark electrode (*Hansatech Oxygraph+*). Measurements were performed at 37 °C using 1 μL of isolated membranes in a 1 mL final volume of the membrane resuspension buffer (25 mM HEPES pH 7.5, 150 mM NaCl). The reaction was started after 30 s of baseline collection by addition of 400 μM NADH. To calculate the rate of the oxygen consumption, the linear range of the measurements was fitted to a linear function, with data extracted from *n* = 6–10 independent measurements. The final values were corrected for the total protein concentration, measured using a Pierce BCA Protein Assay Kit, and for the NADH dehydrogenase activity using an FeCN assay. To this end, FeCN absorption at 410 nm was followed with a UV–vis spectrophotometer (UH5300 Hitachi). Plastic cuvettes were used with 1 mL final volume of membrane resuspension buffer supplemented with 1 mM FeCN and 0.1 mM NADH. The assay was started by addition of 1 μL of isolated membranes with expressed Nqo constructs and performed at 37 °C. The rate of FeCN absorption decay was calculated based on a linear fit (Figure S18). The sf-GFP fluorescence^63^ was scanned using a Cary Eclipse Fluorescence Spectrophotometer (Agilent Technologies), with excitation wavelengths set at 485 nm, and emission scans recorded from 500 to 600 nm. In this regard, 20 μL of isolated membranes was resuspended into a 500 μL final volume of membrane resuspension buffer. The final values were obtained from the 510 nm emission peak and corrected by the total protein concentration.

### Molecular Dynamics Simulations

Atomistic MD simulations were performed on the isolated antiporter modules Nqo12 and Nqo13, embedded in a POPC/POPG/CDL lipid membrane, adapting previous MD models.^29^ Nqo12 and Nqo13 were extracted from the *T. thermophilus* Complex I (PDB ID: 4HEA),^4^ followed by removal of the amphipathic helix of Nqo12 (residues 516–606). The antiporter modules were embedded in the membrane, hydrated with TIP3P water molecules, and neutralized using 150 mM NaCl. The models resulted in systems with *N* = ∼59,400 (Nqo13) and *N* = ∼98.000 (Nqo12) atoms. In silico substitutions were created using Visual Molecular Dynamics (VMD).^64^ The simulations were run in triplicates for 1000 ns each at *T* = 310 K, *p* = 1 bar, and by using a 2 fs time step together with the CHARMM36m force field. Long-range electrostatics were treated using the Particle Mesh Ewald approach with a grid separation of 1 Å. The MD simulations were performed using NAMDv.2.14/v.3.0,^65^ whereas VMD^64^ and MDAnalysis were used for visualization and analysis. See Tables S1 and S2 for further simulations details.^66,67^

### Analysis of Proton Pathways

A structure-based tunnel search algorithm, as implemented in Caver3.0,^69^ was used to identify proton pathways. Heavy atoms were approximated as spheres by their van der Waals radii and searching for the largest cavities using a user-specified starting point toward the bulk solvent. The tunnel probe radius was set to 0.9 Å, with the starting point of the search set at K329 in Nqo12 and at K235 in Nqo13, while excluding protonatable residues as well as bulky gating residue along the channels. The initial tunnels were manually optimized by extending the tunnel to the bulk solvent, combining tunnel segments to obtain connected regions across the complete membrane segment. The water occupancy was obtained by counting water molecules within a 2 Å radius, around a given tunnel coordinate.^68^ The tunnel occupancy was calculated as the ratio of frames with a water molecule at a given tunnel coordinate to the total number of analyzed frames. The electric field vectors at given tunnel coordinates were quantified by *in-house* scripts in combination with TUPÃ,^69^ by excluding solvent molecules, and using a 2 Å cutoff from the measurement point and visualized using VMD.^64^

## Supplementary Material



## Notes

Additional analysis of electric fields, hydration profiles, conformational dynamics of the WT and in silico variants of the antiporter modules, information on simulation parameters, as well as experimental details of the protein expression and purification, calibration of spectroscopic dye molecules, O_2_ consumption data, and details on the data fitting (PDF)

## References

[1] Kaila V. R. I., Wikström M. (2021). Architecture of Bacterial Respiratory
Chains. Nat. Rev. Microbiol..

[2] Hirst J. (2013). Mitochondrial
Complex I. Annu. Rev. Biochem..

[3] Zhu J., Vinothkumar K. R., Hirst J. (2016). Structure of Mammalian Respiratory
Complex I. Nature.

[4] Baradaran R., Berrisford J. M., Minhas G. S., Sazanov L. A. (2013). Crystal
Structure
of the Entire Respiratory Complex I. Nature.

[5] Fiedorczuk K., Letts J. A., Degliesposti G., Kaszuba K., Skehel M., Sazanov L. A. (2016). Atomic Structure
of the Entire Mammalian Mitochondrial
Complex I. Nature.

[6] Grba D. N., Wright J. J., Yin Z., Fisher W., Hirst J. (2024). Molecular
Mechanism of the Ischemia-Induced Regulatory Switch in Mammalian Complex
I. Science.

[7] Kampjut D., Sazanov L. A. (2020). The Coupling Mechanism of Mammalian
Respiratory Complex
I. Science.

[8] Cabrera-Orefice A., Yoga E. G., Wirth C., Siegmund K., Zwicker K., Guerrero-Castillo S., Zickermann V., Hunte C., Brandt U. (2018). Locking Loop
Movement in the Ubiquinone Pocket of Complex I Disengages the Proton
Pumps. Nat. Commun..

[9] Euro L., Belevich G., Verkhovsky M. I., Wikström M., Verkhovskaya M. (2008). Conserved Lysine Residues of the
Membrane Subunit NuoM
Are Involved in Energy Conversion by the Proton-Pumping NADH:Ubiquinone
Oxidoreductase (Complex I). Biochim. Biophys.
Acta BBA - Bioenerg.

[10] Mühlbauer M. E., Saura P., Nuber F., Di Luca A., Friedrich T., Kaila V. R. I. (2020). Water-Gated Proton Transfer Dynamics
in Respiratory
Complex I. J. Am. Chem. Soc..

[11] Dröse S., Krack S., Sokolova L., Zwicker K., Barth H.-D., Morgner N., Heide H., Steger M., Nübel E., Zickermann V., Kerscher S., Brutschy B., Radermacher M., Brandt U. (2011). Functional Dissection of the Proton
Pumping Modules
of Mitochondrial Complex I. PLOS Biol..

[12] Beghiah A., Saura P., Kovalova T., Hoeser F., Friedrich T., Kaila V. R. I. (2026). A Carboxylate
Switch Point Controls Long-Range Energy
Transduction in Respiratory Complex I. openRxiv.

[13] Verkhovskaya M. L., Belevich N., Euro L., Wikström M., Verkhovsky M. I. (2008). Real-Time Electron Transfer in Respiratory
Complex
I. Proc. Natl. Acad. Sci. U. S. A..

[14] Di
Luca A., Gamiz-Hernandez A. P., Kaila V. R. I. (2017). Symmetry-Related
Proton Transfer Pathways in Respiratory Complex I. Proc. Natl. Acad. Sci. U. S. A..

[15] Parey K., Lasham J., Mills D. J., Djurabekova A., Haapanen O., Yoga E. G., Xie H., Kühlbrandt W., Sharma V., Vonck J., Zickermann V. (2021). High-Resolution
Structure and Dynamics of Mitochondrial Complex I—Insights
into the Proton Pumping Mechanism. Sci. Adv..

[16] Haapanen O., Sharma V. (2017). Role of Water and Protein
Dynamics in Proton Pumping
by Respiratory Complex I. Sci. Rep..

[17] Röpke M., Saura P., Riepl D., Pöverlein M. C., Kaila V. R. I. (2020). Functional Water Wires Catalyze Long-Range Proton Pumping
in the Mammalian Respiratory Complex I. J. Am.
Chem. Soc..

[18] Kravchuk V., Petrova O., Kampjut D., Wojciechowska-Bason A., Breese Z., Sazanov L. (2022). A Universal Coupling Mechanism of
Respiratory Complex I. Nature.

[19] Michel J., DeLeon-Rangel J., Zhu S., Van Ree K., Vik S. B. (2011). Mutagenesis
of the L, M, and N Subunits of Complex I from Escherichia Coli Indicates
a Common Role in Function. PLoS One.

[20] Nakamaru-Ogiso E., Kao M.-C., Chen H., Sinha S. C., Yagi T., Ohnishi T. (2010). The Membrane Subunit
NuoL­(ND5) Is Involved in the Indirect
Proton Pumping Mechanism of Escherichia Coli Complex I. J. Biol. Chem..

[21] Jarman O. D., Hirst J. (2022). Membrane-Domain Mutations in Respiratory Complex I Impede Catalysis
but Do Not Uncouple Proton Pumping from Ubiquinone Reduction. PNAS Nexus.

[22] Amarneh B., Vik S. B. (2003). Mutagenesis of Subunit
N of the Escherichia Coli Complex
I. Identification of the Initiation Codon and the Sensitivity of Mutants
to Decylubiquinone. Biochemistry.

[23] Kaila V. R. I. (2018). Long-Range
Proton-Coupled Electron Transfer in Biological Energy Conversion:
Towards Mechanistic Understanding of Respiratory Complex I. J. R. Soc. Interface.

[24] Kaila V. R. I. (2021). Resolving
Chemical Dynamics in Biological Energy Conversion: Long-Range Proton-Coupled
Electron Transfer in Respiratory Complex I. Acc. Chem. Res..

[25] Gamiz-Hernandez A. P., Jussupow A., Johansson M. P., Kaila V. R. I. (2017). Terminal Electron–Proton
Transfer Dynamics in the Quinone Reduction of Respiratory Complex
I. J. Am. Chem. Soc..

[26] Tocilescu M. A., Fendel U., Zwicker K., Kerscher S., Brandt U. (2007). Exploring
the Ubiquinone Binding Cavity of Respiratory Complex I. J. Biol. Chem..

[27] Sharma V., Belevich G., Gamiz-Hernandez A. P., Róg T., Vattulainen I., Verkhovskaya M. L., Wikström M., Hummer G., Kaila V. R. I. (2015). Redox-Induced Activation of the Proton
Pump in the Respiratory Complex I. Proc. Natl.
Acad. Sci. U. S. A..

[28] Kim H., Saura P., Pöverlein M. C., Gamiz-Hernandez A. P., Kaila V. R. I. (2023). Quinone Catalysis Modulates Proton Transfer Reactions
in the Membrane Domain of Respiratory Complex I. J. Am. Chem. Soc..

[29] Beghiah A., Saura P., Badolato S., Kim H., Zipf J., Auman D., Gamiz-Hernandez A. P., Berg J., Kemp G., Kaila V. R. I. (2024). Dissected Antiporter Modules Establish Minimal Proton-Conduction
Elements of the Respiratory Complex I. Nat.
Commun..

[30] Grba D. N., Hirst J. (2020). Mitochondrial Complex
I Structure Reveals Ordered Water Molecules
for Catalysis and Proton Translocation. Nat.
Struct. Mol. Biol..

[31] Warnau J., Sharma V., Gamiz-Hernandez A. P., Di Luca A., Haapanen O., Vattulainen I., Wikström M., Hummer G., Kaila V. R. I. (2018). Redox-Coupled
Quinone Dynamics in the Respiratory Complex I. Proc. Natl. Acad. Sci. U. S. A..

[32] Gupta C., Khaniya U., Chan C. K., Dehez F., Shekhar M., Gunner M. R., Sazanov L., Chipot C., Singharoy A. (2020). Charge Transfer
and Chemo-Mechanical Coupling in Respiratory Complex I. J. Am. Chem. Soc..

[33] Haapanen O., Djurabekova A., Sharma V. (2019). Role of Second Quinone Binding Site
in Proton Pumping by Respiratory Complex I. Front. Chem..

[34] Bridges H. R., Fedor J. G., Blaza J. N., Di Luca A., Jussupow A., Jarman O. D., Wright J. J., Agip A.-N. A., Gamiz-Hernandez A. P., Roessler M. M., Kaila V. R. I., Hirst J. (2020). Structure of Inhibitor-Bound
Mammalian Complex I. Nat. Commun..

[35] Klusch N., Dreimann M., Senkler J., Rugen N., Kühlbrandt W., Braun H.-P. (2023). Cryo-EM Structure of the Respiratory
I + III2 Supercomplex
from Arabidopsis Thaliana at 2 Å Resolution. Nat. Plants.

[36] Pohl P., Saparov S. M., Borgnia M. J., Agre P. (2001). Highly Selective Water
Channel Activity Measured by Voltage Clamp: Analysis of Planar Lipid
Bilayers Reconstituted with Purified AqpZ. Proc.
Natl. Acad. Sci. U. S. A..

[37] Kratochvil H. T., Watkins L. C., Mravic M., Thomaston J. L., Nicoludis J. M., Somberg N. H., Liu L., Hong M., Voth G. A., DeGrado W. F. (2023). Transient Water Wires Mediate Selective
Proton Transport in Designed Channel Proteins. Nat. Chem..

[38] Hoeser F., Tausend H., Götz S., Wohlwend D., Einsle O., Günther S., Friedrich T. (2022). Respiratory Complex I with Charge
Symmetry in the Membrane Arm Pumps Protons. Proc. Natl. Acad. Sci. U. S. A..

[39] Kao M.-C., Nakamaru-Ogiso E., Matsuno-Yagi A., Yagi T. (2005). Characterization of
the Membrane Domain Subunit NuoK (ND4L) of the NADH-Quinone Oxidoreductase
from Escherichia Coli. Biochemistry.

[40] Pöverlein M. C., Hulm A., Dietschreit J. C. B., Kussmann J., Ochsenfeld C., Kaila V. R. I. (2024). QM/MM Free Energy
Calculations of Long-Range Biological
Protonation Dynamics by Adaptive and Focused Sampling. J. Chem. Theory Comput..

[41] Zdorevskyi O., Djurabekova A., Lasham J., Sharma V. (2023). Horizontal
Proton Transfer
across the Antiporter-like Subunits in Mitochondrial Respiratory Complex
I. Chem. Sci..

[42] Allgöwer F., Gamiz-Hernandez A. P., Rutherford A. W., Kaila V. R. I. (2022). Molecular Principles
of Redox-Coupled Protonation Dynamics in Photosystem II. J. Am. Chem. Soc..

[43] Allgöwer F., Pöverlein M. C., Rutherford A. W., Kaila V. R. I. (2024). Mechanism of
Proton Release during Water Oxidation in Photosystem II. Proc. Natl. Acad. Sci. U. S. A..

[44] Saura P., Riepl D., Frey D. M., Wikström M., Kaila V. R. I. (2022). Electric Fields Control Water-Gated Proton Transfer
in Cytochrome c Oxidase. Proc. Natl. Acad. Sci.
U. S. A..

[45] Pisliakov A. V., Sharma P. K., Chu Z. T., Haranczyk M., Warshel A. (2008). Electrostatic Basis for the Unidirectionality of the
Primary Proton Transfer in Cytochrome c Oxidase. Proc. Natl. Acad. Sci. U. S. A..

[46] Peng Y., Swanson J. M. J., Kang S., Zhou R., Voth G. A. (2015). Hydrated
Excess Protons Can Create Their Own Water Wires. J. Phys. Chem. B.

[47] Liang R., Swanson J. M. J., Wikström M., Voth G. A. (2017). Understanding the
Essential Proton-Pumping Kinetic Gates and Decoupling Mutations in
Cytochrome c Oxidase. Proc. Natl. Acad. Sci.
U. S. A..

[48] Saura P., Frey D. M., Gamiz-Hernandez A. P., Kaila V. R. I. (2019). Electric Field
Modulated Redox-Driven Protonation and Hydration Energetics in Energy
Converting Enzymes. Chem. Commun..

[49] Son C. Y., Yethiraj A., Cui Q. (2017). Cavity Hydration
Dynamics in Cytochrome *c* Oxidase and Functional Implications. Proc. Natl. Acad. Sci. U. S. A..

[50] Godoy-Hernandez A., Asseri A. H., Purugganan A. J., Jiko C., de Ram C., Lill H., Pabst M., Mitsuoka K., Gerle C., Bald D., McMillan D. G. G. (2023). Rapid
and Highly Stable Membrane
Reconstitution by LAiR Enables the Study of Physiological Integral
Membrane Protein Functions. ACS Cent. Sci..

[51] Kano K., Fendler J. H. (1978). Pyranine as a Sensitive
pH Probe for Liposome Interiors
and Surfaces. pH Gradients across Phospholipid Vesicles. Biochim. Biophys. Acta BBA - Biomembr..

[52] Bashford C. L., Thayer W. S. (1977). Thermodynamics of
the Electrochemical Proton Gradient
in Bovine Heart Submitochondrial Particles. J. Biol. Chem..

[53] Kaila V. R. I., Verkhovsky M. I., Wikström M. (2010). Proton-Coupled
Electron Transfer in Cytochrome Oxidase. Chem.
Rev..

[54] Kaila V. R.
I., Verkhovsky M. I., Hummer G., Wikström M. (2008). Glutamic Acid
242 Is a Valve in the Proton Pump of Cytochrome c Oxidase. Proc. Natl. Acad. Sci. U. S. A..

[55] Deutschmann S., Täuber S. T., Rimle L., Biner O., Schori M., Stanic A.-M., von Ballmoos C. (2024). Modulating
Liposome Surface Charge
for Maximized ATP Regeneration in Synthetic Nanovesicles. ACS Synth. Biol..

[56] Henzler-Wildman K. A., Lei M., Thai V., Kerns S. J., Karplus M., Kern D. (2007). A Hierarchy
of Timescales in Protein Dynamics Is Linked to Enzyme Catalysis. Nature.

[57] Kaila V. R. I., Wikström M., Hummer G. (2014). Electrostatics, Hydration,
and Proton Transfer Dynamics in the Membrane Domain of Respiratory
Complex I. Proc. Natl. Acad. Sci. U. S. A..

[58] Zhang X. C., Li B. (2019). Towards Understanding
the Mechanisms of Proton Pumps in Complex-I
of the Respiratory Chain. Biophys. Rep..

[59] Djurabekova A., Lasham J., Zdorevskyi O., Zickermann V., Sharma V. (2024). Long-Range Electron Proton Coupling
in Respiratory
Complex I—Insights from Molecular Simulations of the Quinone
Chamber and Antiporter-like Subunits. Biochem.
J..

[60] Schiller J., Laube E., Wittig I., Kühlbrandt W., Vonck J., Zickermann V. (2022). Insights into
Complex I Assembly:
Function of NDUFAF1 and a Link with Cardiolipin Remodeling. Sci. Adv..

[61] Drew D. E., von Heijne G., Nordlund P., de Gier J.-W. L. (2001). Green Fluorescent
Protein as an Indicator to Monitor Membrane Protein Overexpression
in Escherichia Coli. FEBS Lett..

[62] Hjelm, A. ; Schlegel, S. ; Baumgarten, T. ; Klepsch, M. ; Wickström, D. ; Drew, D. ; de Gier, J.-W. O. E. Coli-Based Membrane Protein Production Using Lemo21­(DE3) and GFP-Fusions. In Membrane Biogenesis: Methods and Protocols; Rapaport, D. , Herrmann, J. M. , Eds.; Humana Press: Totowa, NJ, 2013; pp 381–400. 10.1007/978-1-62703-487-6_24. 23996190

[63] Drew D., Newstead S., Sonoda Y., Kim H., von Heijne G., Iwata S. (2008). GFP-Based Optimization Scheme for
the Overexpression and Purification
of Eukaryotic Membrane Proteins in Saccharomyces Cerevisiae. Nat. Protoc..

[64] Humphrey W., Dalke A., Schulten K. V. M. D. (1996). Visual Molecular Dynamics. J. Mol. Graph..

[65] Phillips J. C., Hardy D. J., Maia J. D. C., Stone J. E., Ribeiro J. V., Bernardi R. C., Buch R., Fiorin G., Hénin J., Jiang W., McGreevy R., Melo M. C. R., Radak B. K., Skeel R. D., Singharoy A., Wang Y., Roux B., Aksimentiev A., Luthey-Schulten Z., Kalé L. V., Schulten K., Chipot C., Tajkhorshid E. (2020). Scalable Molecular
Dynamics on CPU and GPU Architectures with NAMD. J. Chem. Phys..

[66] Gowers, R. J. ; Linke, M. ; Barnoud, J. ; Reddy, T. J. E. ; Melo, M. N. ; Seyler, S. L. ; Domanski, J. ; Dotson, D. L. ; Buchoux, S. ; Kenney, I. M. ; Beckstein, O. MDAnalysis: A Python Package for the Rapid Analysis of Molecular Dynamics Simulations. In Report Number: LA-UR-19–29136; Research Org.; Los Alamos National Laboratory (LANL) 2019.11 AD.

[67] Michaud-Agrawal N., Denning E. J., Woolf T. B., Beckstein O. (2011). MDAnalysis:
A Toolkit for the Analysis of Molecular Dynamics Simulations. J. Comput. Chem..

[68] Chovancova E., Pavelka A., Benes P., Strnad O., Brezovsky J., Kozlikova B., Gora A., Sustr V., Klvana M., Medek P., Biedermannova L., Sochor J., Damborsky J. (2012). CAVER 3.0:
A Tool for the Analysis of Transport Pathways in Dynamic Protein Structures. PLOS Comput. Biol..

[69] Polêto M. D., Lemkul J. A. (2022). TUPÃ: Electric Field Analyses
for Molecular
Simulations. J. Comput. Chem..

