# Supplementary material for: Molecular Principles of Gating Proton Transport in the Antiporter Modules of Respiratory Complex I
Source: J Am Chem Soc. 2026 May 8;148(20):21056–68. doi: 10.1021/jacs.6c05956 (PMC13220270; doi:10.1021/jacs.6c05956)
Supplement: Supplementary file 1 [file ja6c05956_si_001.pdf]

Supplementary Information

for

Molecular Principles of Gating Proton Transport in the Antiporter Modules of Respiratory Complex I

Sofia Badolato[1,a], Sarah Rossmann[1,a], Joana Pereira[1], Hyunho Kim[1], Ville R. I. Kaila[1,*]

[1]Department of Biochemistry and Biophysics, Stockholm University, 10691, Stockholm, Sweden.
[a] Equal contribution. [*]Corresponding author: Ville R. I. Kaila, **E-mail:** ville.kaila@dbb.su.se

Content

Figure S1 Hydration state and conformational dynamics during proton transfer in Nqo12.
Figure S2 Electric field effects upon alteration of protonation states in Nqo12.
Figure S3 Electric field differences upon proton transfer in Nqo12.
Figure S4 Hydration state and conformational dynamics during proton transfer in Nqo13.
Figure S5 Electric field effects upon alteration of protonation states in Nqo13.
Figure S6 Electric field differences upon proton transfer in Nqo13.
Figure S7 Alternative P-side output pathway in Nqo13.
Figure S8 Visualization of electric field effects in antiporter modules.
Figure S9 Hydration state, conformational dynamics, and electric field effects upon *in silico* mutation of Nqo12.
Figure S10 Hydration state, conformational dynamics, and electric field effects upon *in silico* mutation of Nqo13.
Figure S11 Overlay of water structure from MD simulations of Nqo12 and Nqo13.
Figure S12 Central distances and dihedral angles during MD simulations.
Figure S13 Calibration of pyranine and oxonol VI.
Figure S14 Protein expression, purification, and stability.
Figure S15 GFP fluorescence of antiporter modules in membranes.
Figure S16 O_2 consumption in membranes upon expression of the antiporter variants.
Figure S17 Initial rates of proton conduction from proteoliposome experiments.
Figure S18 Data fitting.

Table S1 List of MD simulations.
Table S2 List of base protonation states.
Table S3 List of designed primers.
Table S4 K^+_{in}/K^+_{out} concentrations employed for calibration of $\Delta\psi$.

SI References

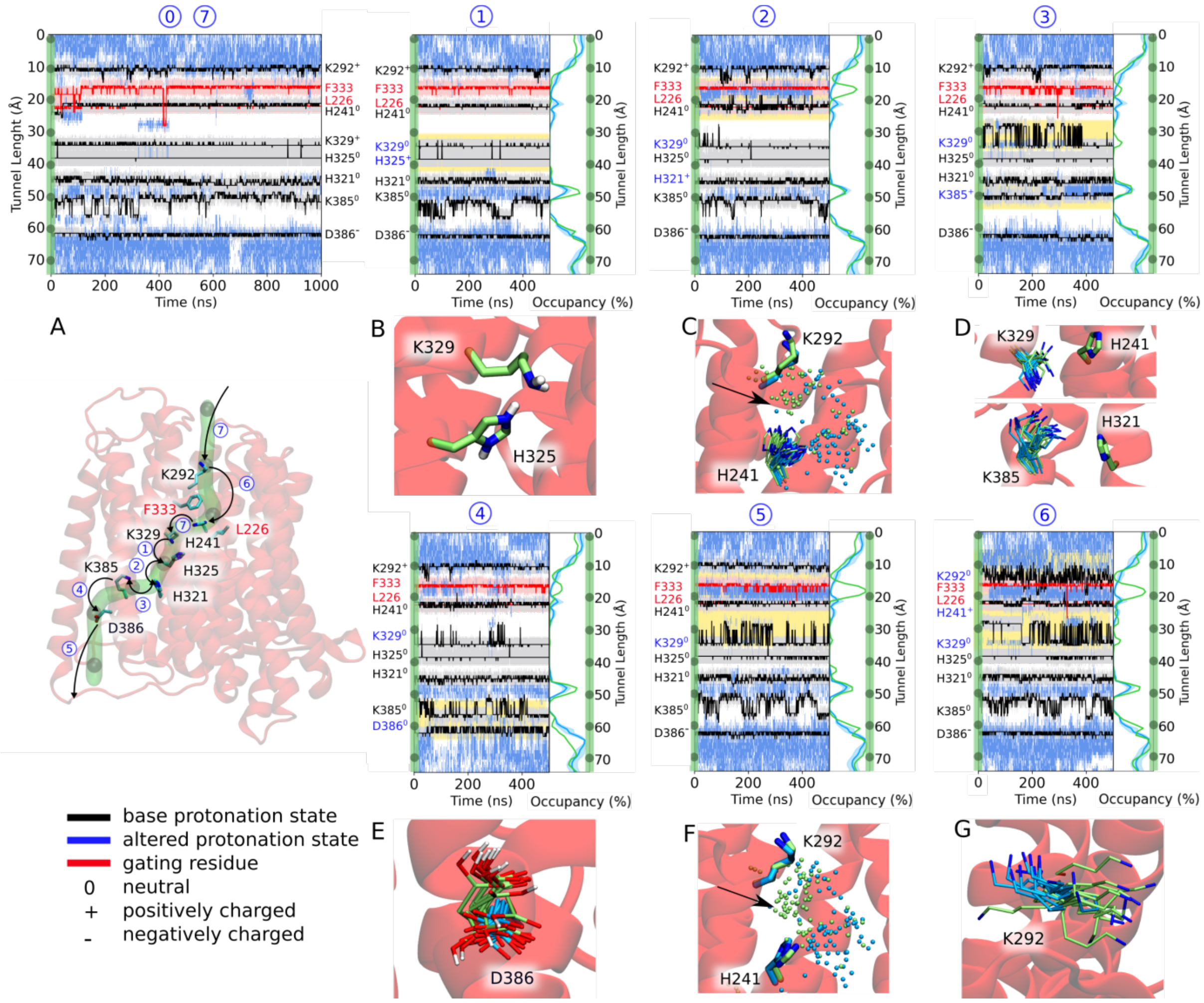

Figure S1. Hydration state and conformational dynamics during proton transfer in Nqo12. (0, 7) Hydration profile during the MD simulation of the base protonation state (see **Table S2**), and **(1-6)** altered protonation states along the proton pathway with the projected position of titratable residues (in black), non-polar gates (in red), hydration event (in blue), projected on the tunnel coordinates. The hydrodynamic radii of residues are shown as semitransparent colors, and regions of special interest are highlighted in yellow (see panel 1-6). **A)** Studied proton transfer reactions, with individual steps marked in 0-7. **B-G)** MD snapshot of regions of interest with base protonation states in blue and altered protonation states in green.

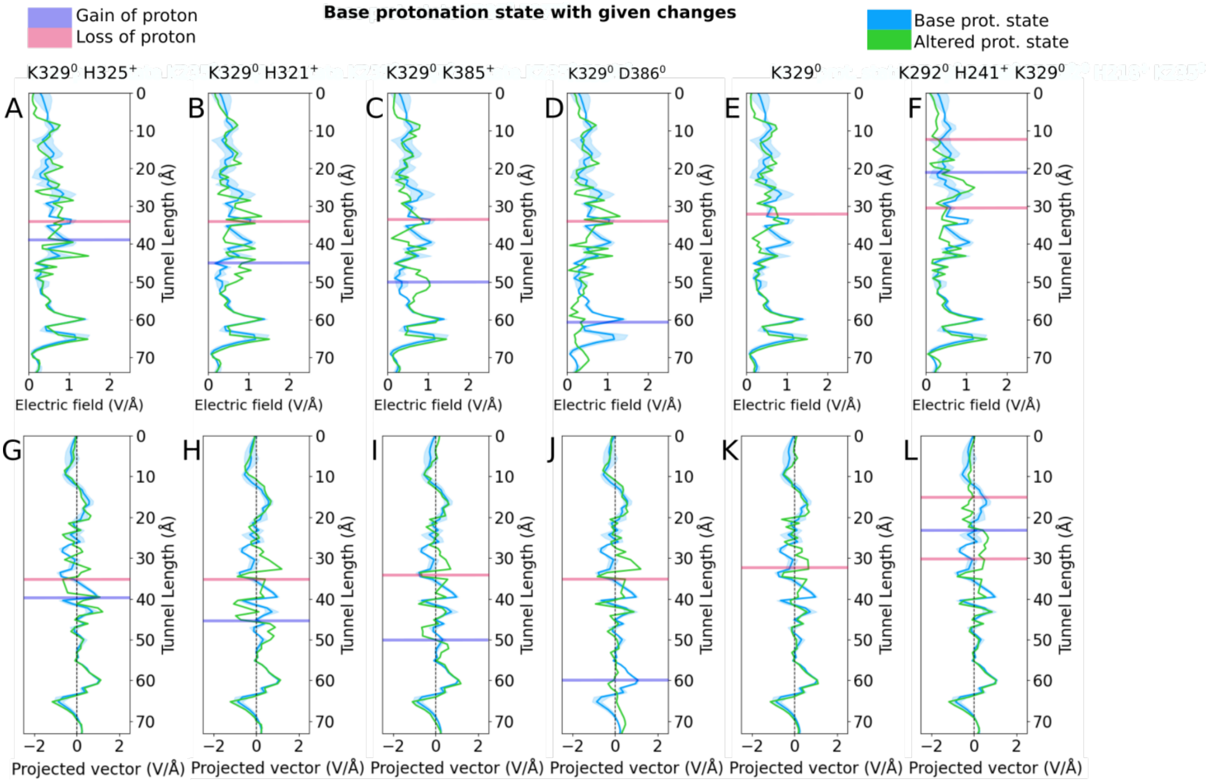

Figure S2. Electric field effects upon alteration of protonation states in Nqo12. A-F) Magnitude of electric field vector along the proton tunnel. **G-L)** Electric field vector projected onto the pathway. The base protonation state is shown in blue and the altered protonation state in green. The position of proton donating residues are marked with a pink line, whereas proton accepting residues are marked with a blue line.

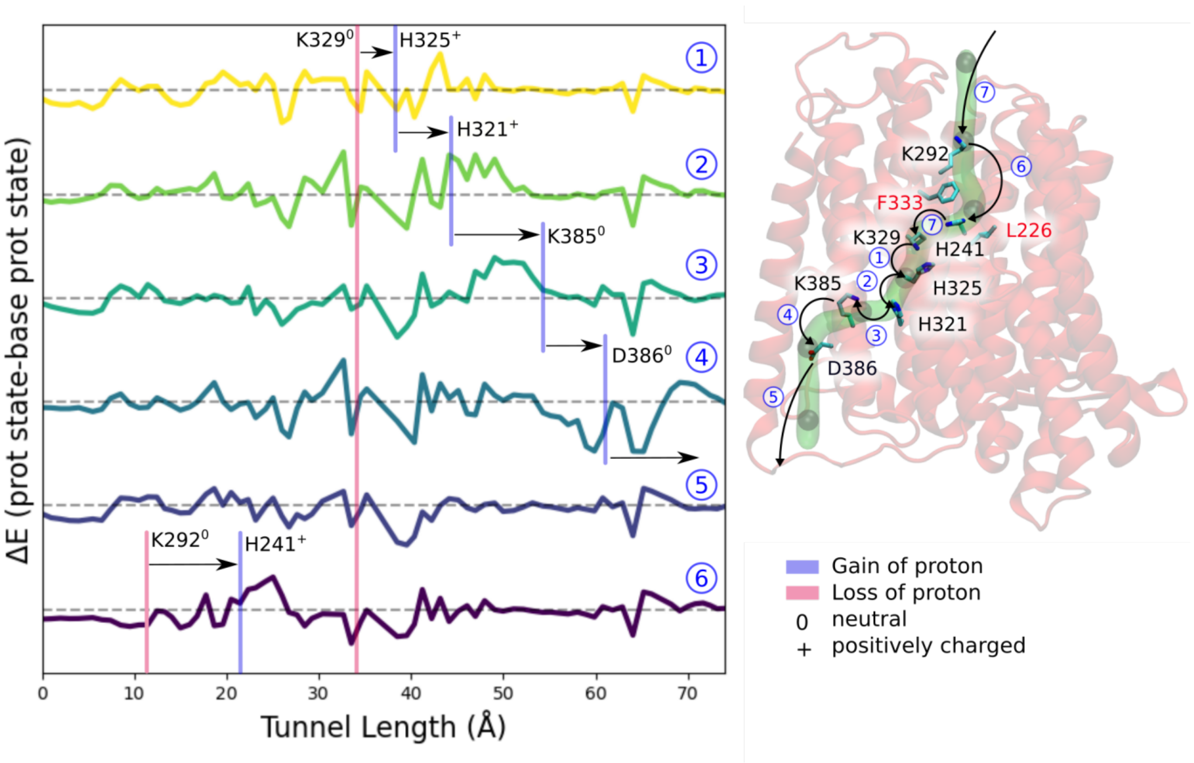

Figure S3. Electric field differences upon proton transfer in Nqo12. The position of proton donating residues are marked with a pink line, whereas proton accepting residues are marked with a blue line. A schematic representation of the modeled proton transfer process is shown on the right.

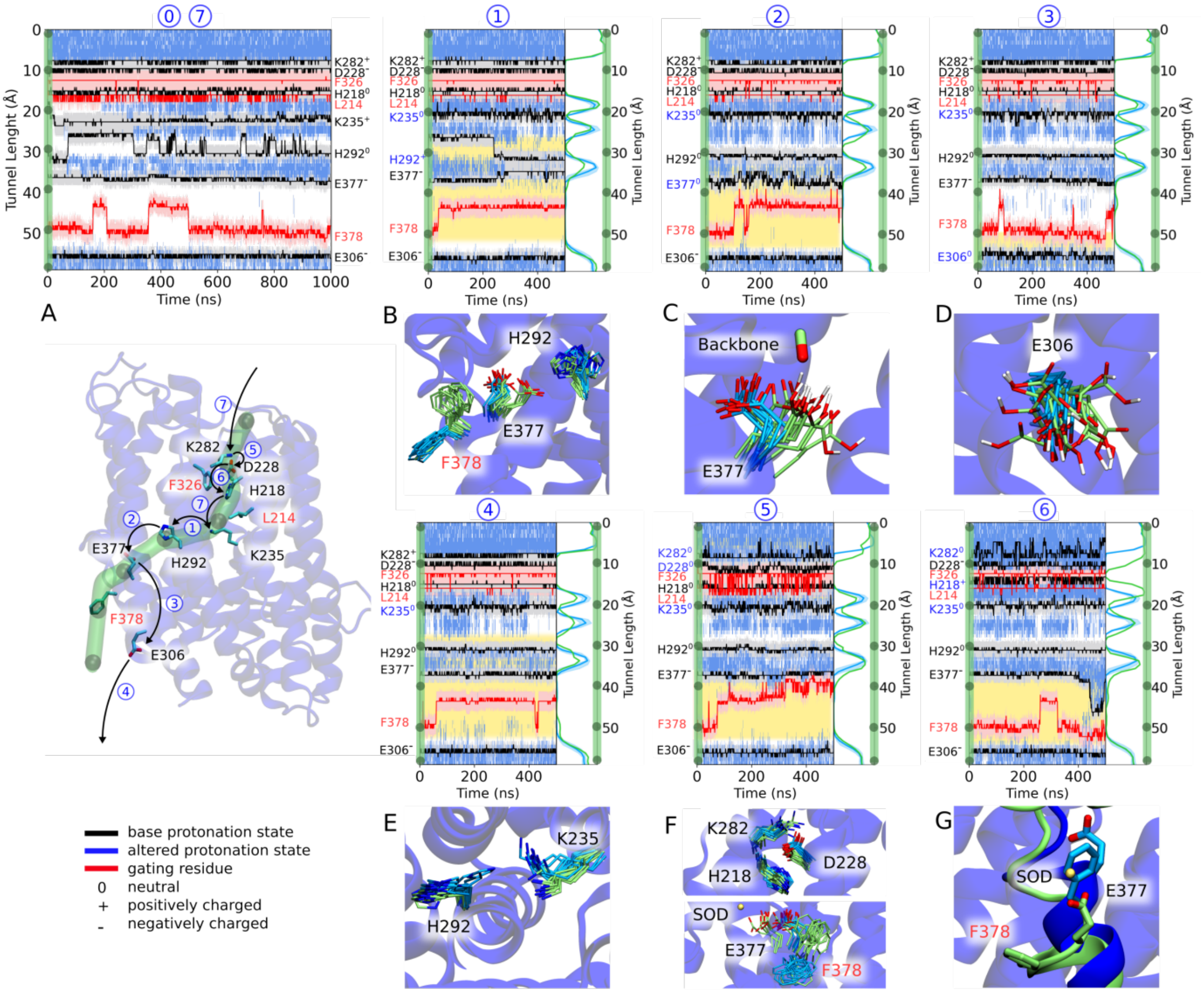

Figure S4. Hydration state and conformational dynamics during proton transfer in Nqo13. (0, 7) Hydration profile during the MD simulation of the base protonation state (see **Table S2**), and **(1-6)** altered protonation states along the proton pathway with projected position of titratable residues (in black), non-polar gates (in red), hydration event (in blue), on the tunnel coordinates. The hydrodynamic radii of residues are shown as semitransparent colors, and regions of special interest are highlighted in yellow (see panel 1-6). **A)** Studied proton transfer reactions, with individual steps marked in 0-7. **B)** MD snapshot of regions of interest with base protonation states in blue and altered protonation states in green.

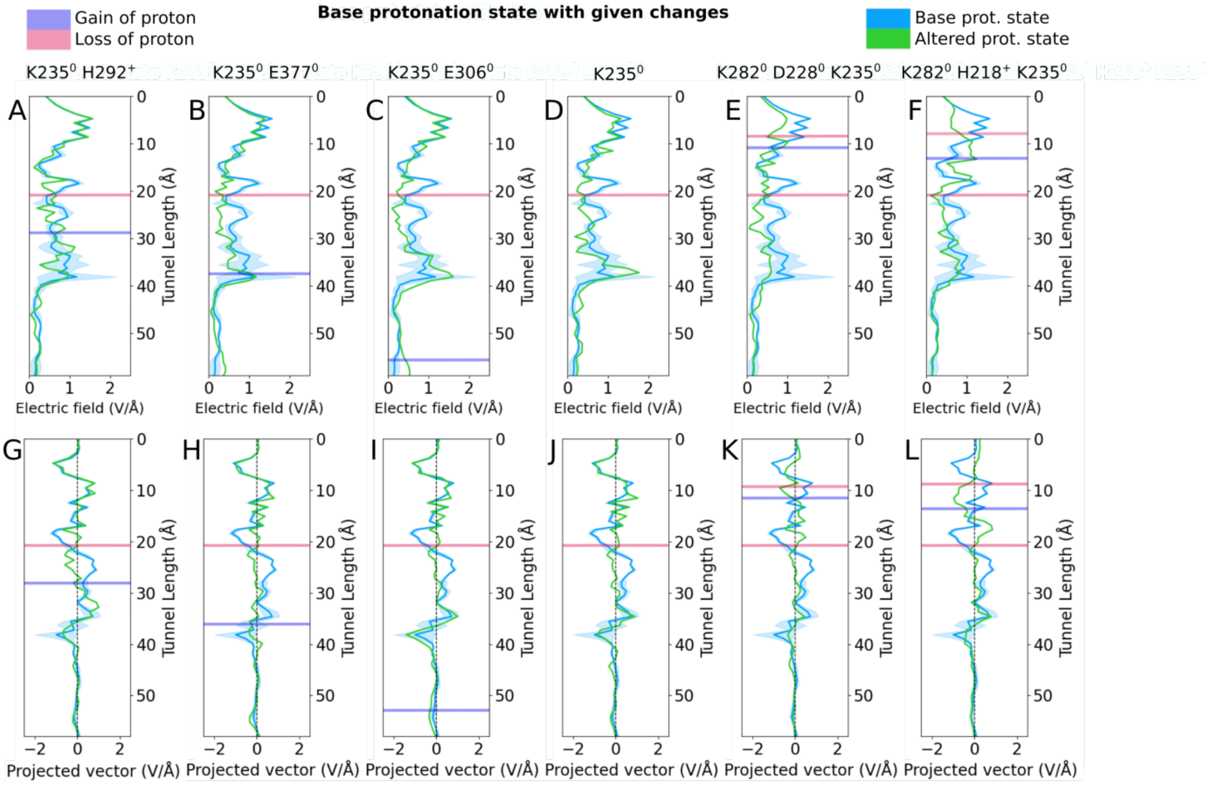

Figure S5. Electric field effects upon alteration of protonation states in Nqo13. A-F) Electric field vector magnitude over tunnel position. **G-L)** electric field vector projected onto proton pathway. The base protonation state is shown in blue and the altered protonation state in green. The position of proton donating residues are marked with a pink line, whereas proton accepting residues are marked with blue line.

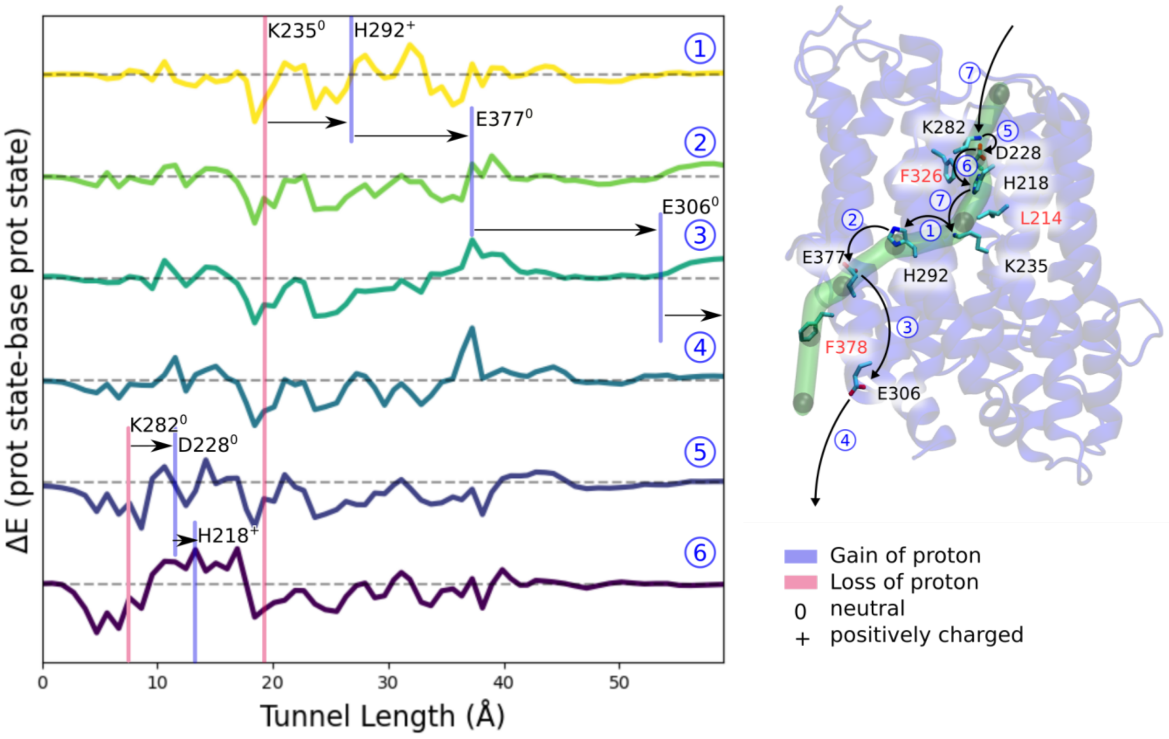

Figure S6. Electric field differences upon proton transfer in Nqo13. The position of proton donating residues are marked with a pink line, whereas proton accepting residues are marked with blue line. A schematic representation of the modeled proton transfer process is shown on the right.

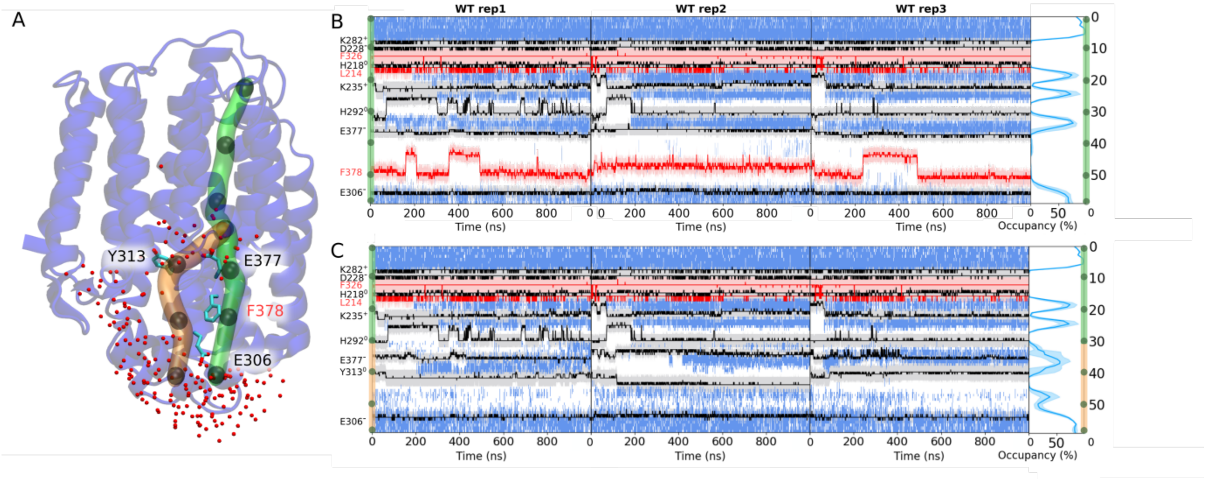

Figure S7. Alternative P-side output pathway in Nqo13. A) Putative proton output pathway shown in green (analogous to P-side pathway in Nqo12) and alternative pathway shown in orange. Key residues along the pathways are shown. Hydration profiles along **B)**, the original, and **C)**, the alternative pathway with water occupancy shown on the right.

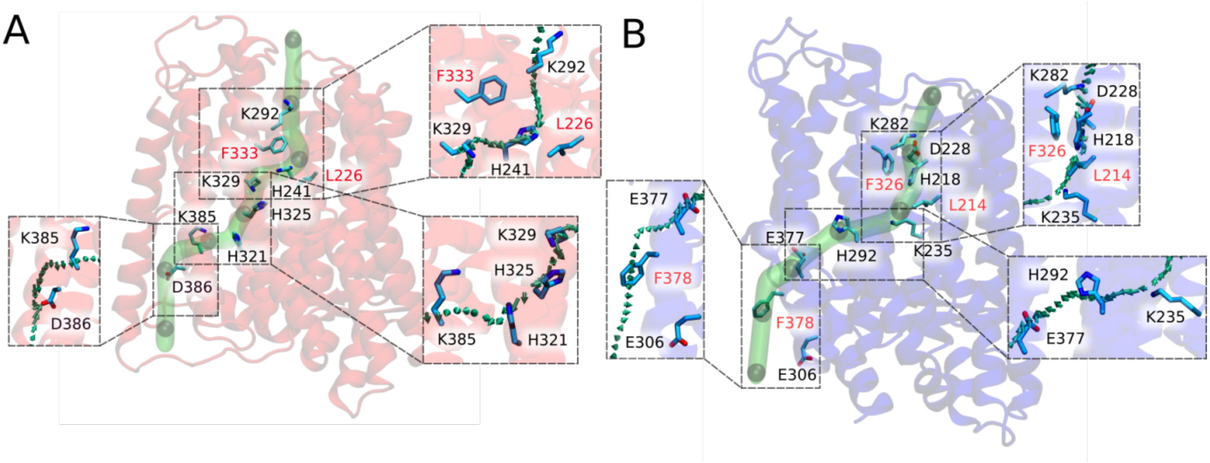

Figure S8. **Visualization of electric field effects in antiporter modules.** Electric field vectors in **A)** Nqo12, and **B)** Nqo13.

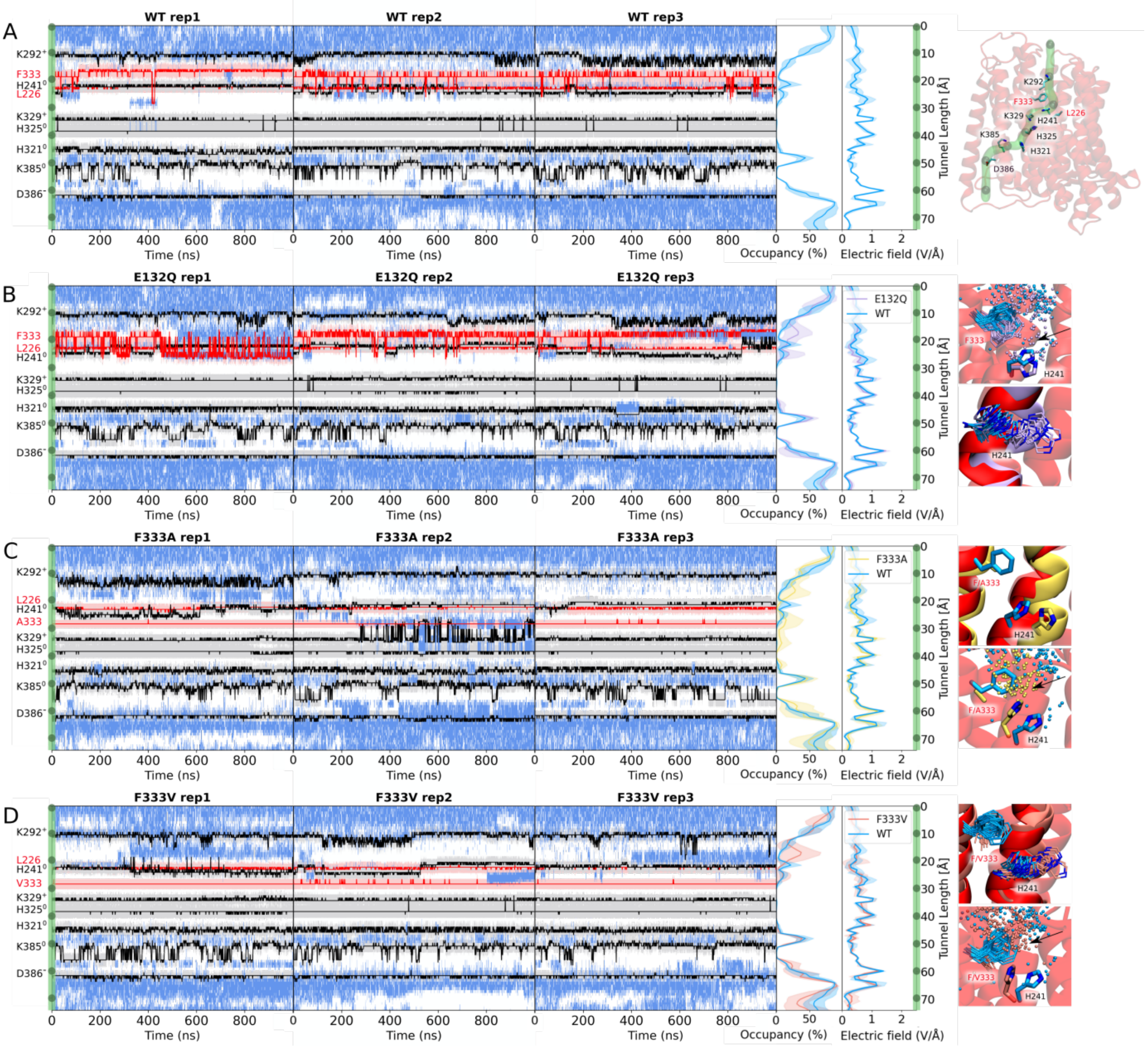

Figure S9. Hydration state, conformational dynamics, and electric field effects upon *in silico* mutation of Nqo12. A-G) Time-dependent hydration profiles during MD simulation (from three replicas) along proton pathway with position of titratable residues (in black), non-polar gates (in red), hydration event (in blue), projected on the tunnel coordinates. The hydrodynamic radii of residues are shown as semitransparent colors. MD snapshots WT (in blue) and mutant (colored). The average water occupancy and electric field strength along the pathway are averaged over triplicates, with error bars (standard deviations) marked as shaded areas. Right inset: MD snapshots comparing WT (blue), and mutant (colored).

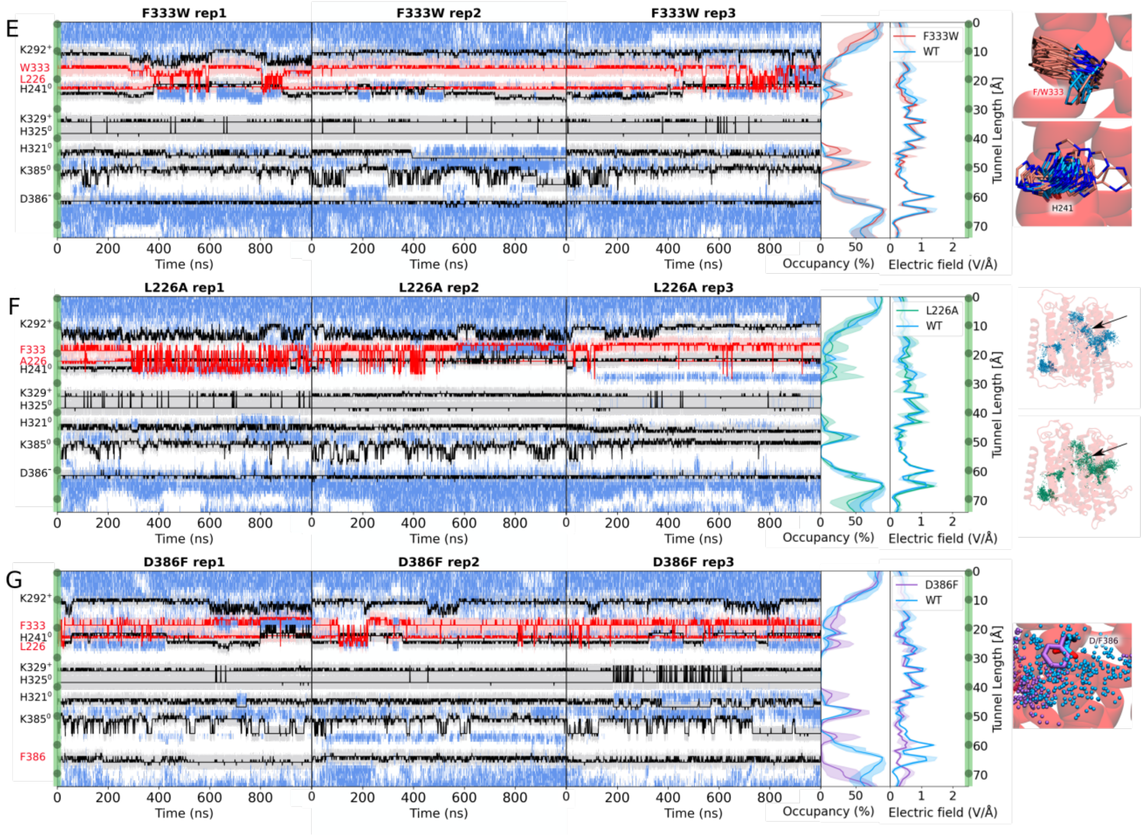

Figure S9 (continued) Hydration state, conformational dynamics, and electric field effects upon *in silico* mutation of Nqo12. A-G) Time-dependent hydration profiles during MD simulation (from three replicas) along proton pathway with position of titratable residues (in black), non-polar gates (in red), hydration event (in blue), projected on the tunnel coordinates. The hydrodynamic radii of residues are shown as semitransparent colors. MD snapshots WT (in blue) and mutant (colored). The average water occupancy and electric field strength along the pathway are averaged over triplicates, with error bars (standard deviations) marked as shaded areas. Right inset: MD snapshots comparing WT (blue), and mutant (colored).

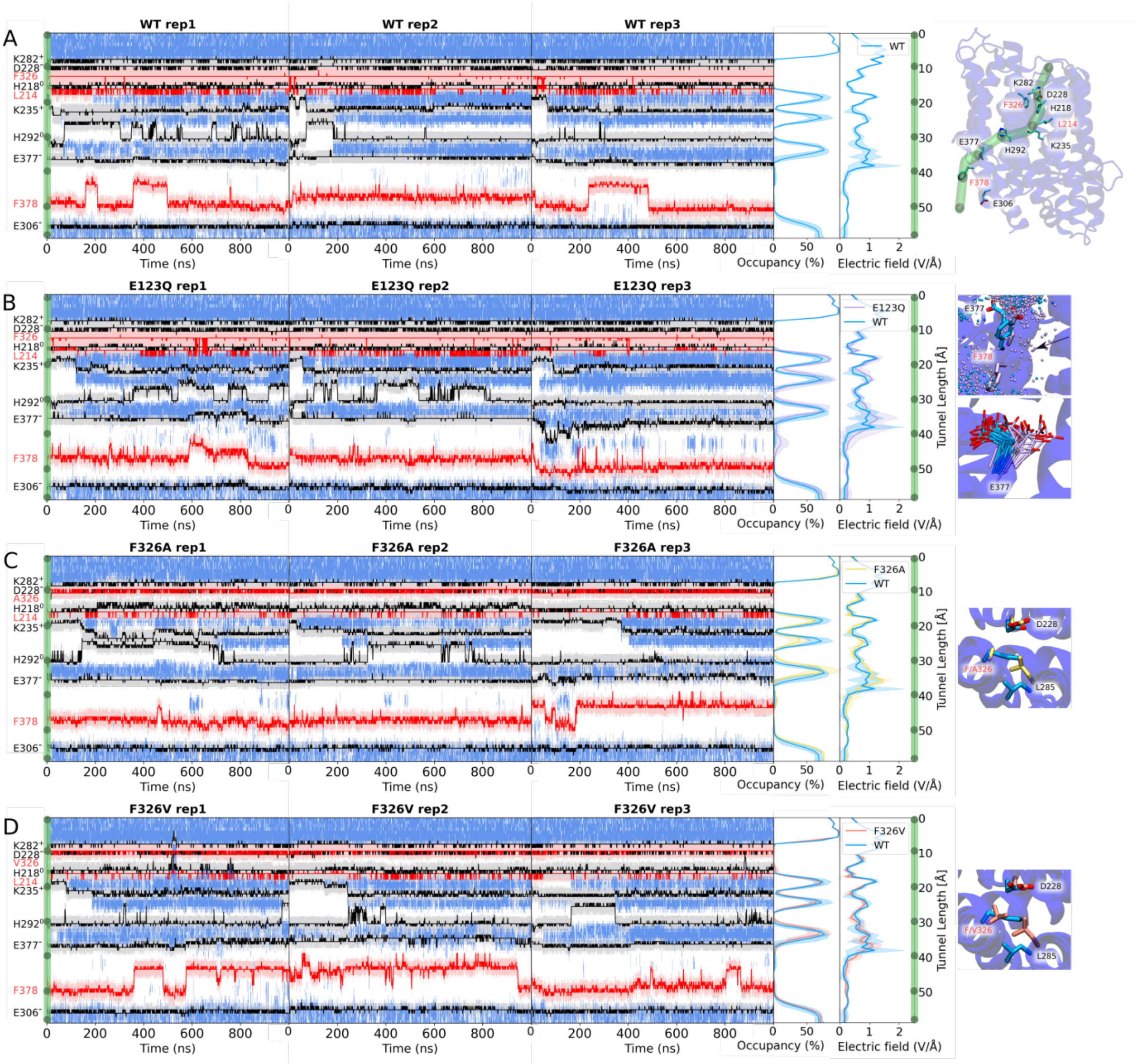

Figure S10. Hydration state, conformational dynamics, and electric field effects upon *in silico* mutation of Nqo13. A-G) Time-dependent hydration profiles during MD simulation (for three replicas) along proton pathway with position of titratable residues (in black), non-polar gates (in red), hydration event (in blue), projected on the tunnel coordinates. The hydrodynamic radii of residues are shown as semitransparent colors. MD snapshots WT (in blue) and mutant (colored). The average water occupancy and electric field strength along the pathway are averaged over triplicates, with error bars (standard deviations) marked as shaded areas. Right inset: MD snapshots comparing WT (blue) and mutant (colored).

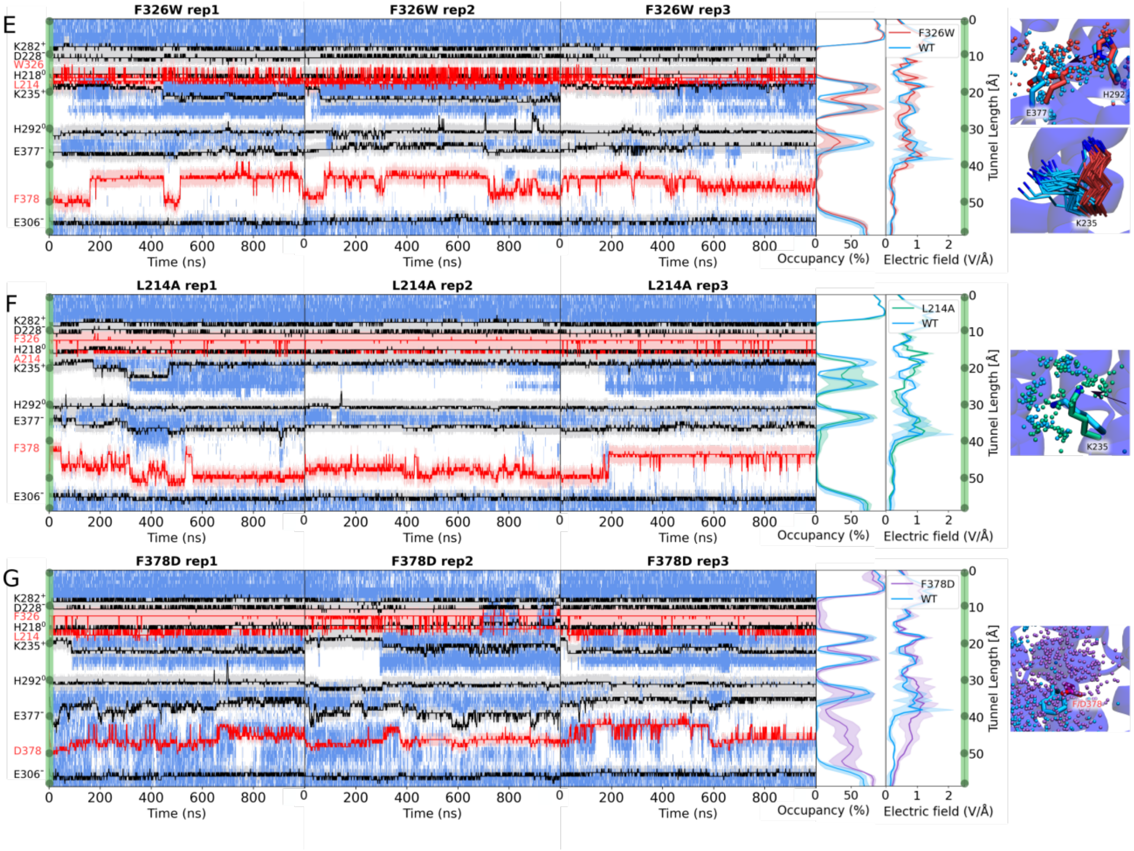

Figure S10 (continued) Hydration state, conformational dynamics, and electric field effects upon *in silico* mutation of Nqo13. A-G) Time-dependent hydration profiles during MD simulation (for three replicas) along proton pathway with project position of titratable residues (in black), non-polar gates (in red), hydration event (in blue), projected on the tunnel coordinate. The hydrodynamic radii of residues are shown as semitransparent colors. MD snapshots WT (in blue) and mutant (colored). The average water occupancy and electric field strength along the pathway are averaged over triplicates, with error bars (standard deviations) marked as shaded areas. Right inset: MD snapshots comparing WT (blue) and mutant (colored).

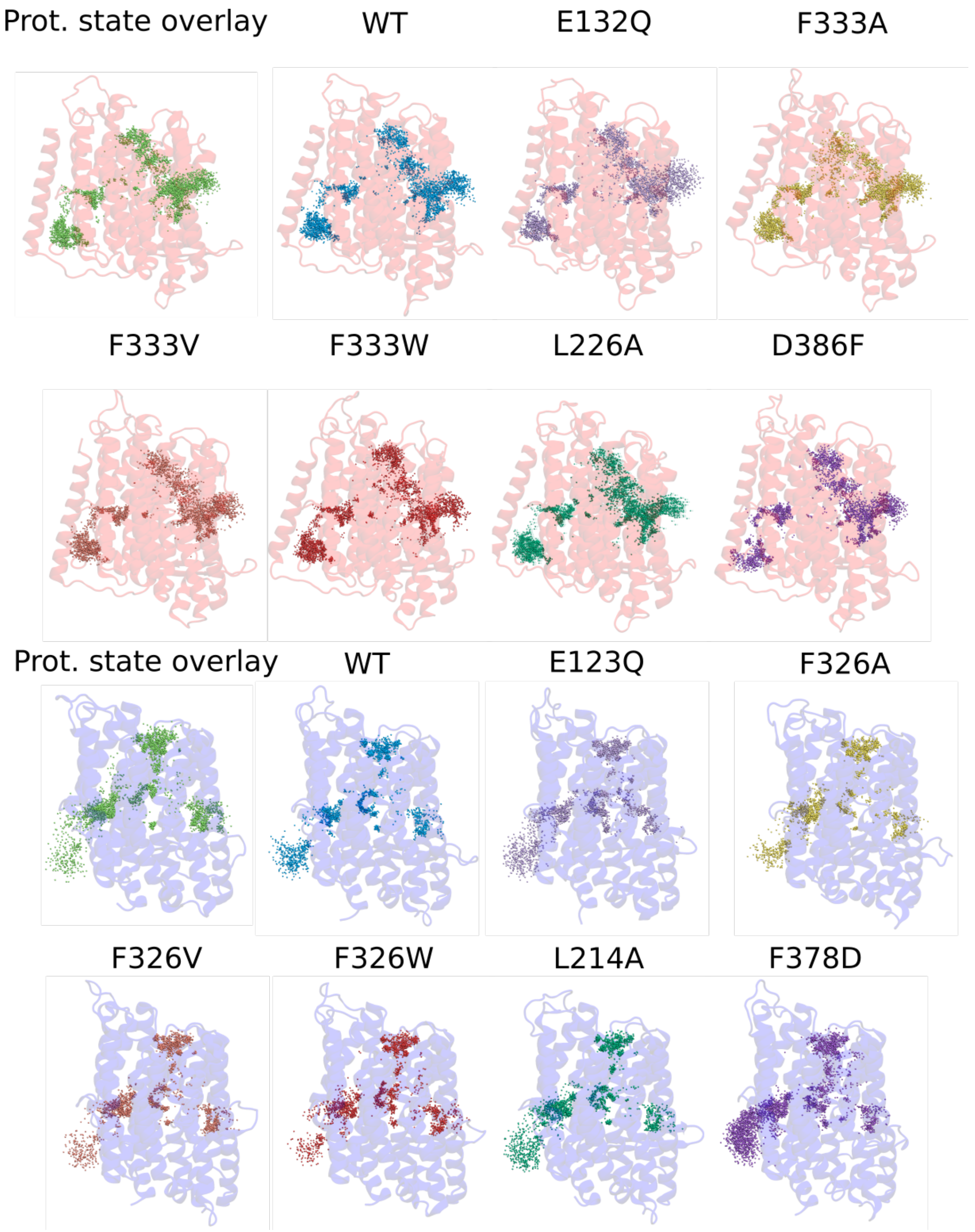

Figure S11. Overlay of water structure from MD simulations of Nqo12 and Nqo13. The water structure is averaged over three replicas (**Table S1**).

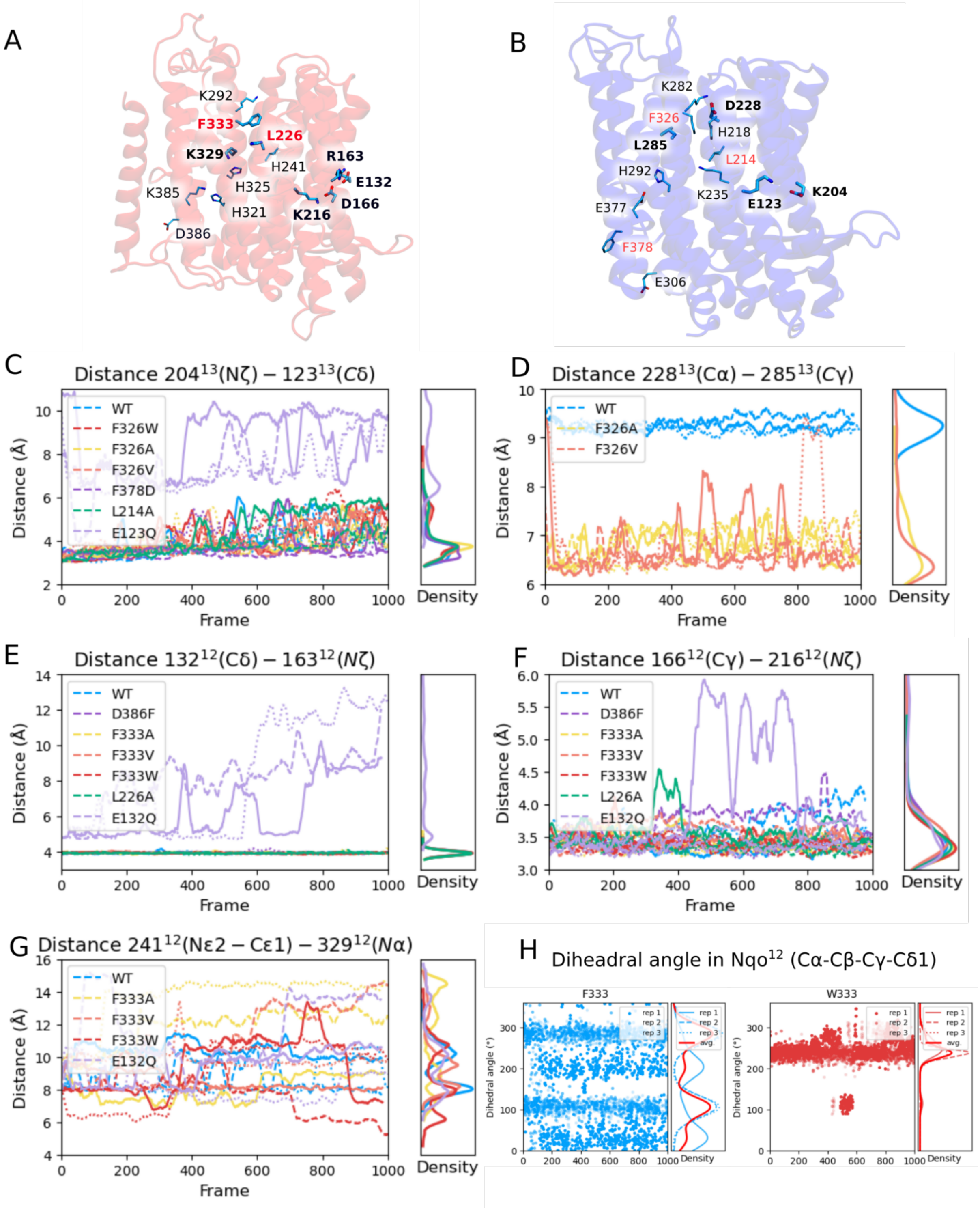

Figure S12. Central distances and dihedral angles during MD simulations. A) Structure of Nqo12 and **B)** Nqo13 with key residues shown as sticks. **C)** Ion-pair distance and **D)** distance between residues 228-285, highlighting a structural change in F326A/V of Nqo13. **E,F)** Ion-pair distances and **G)** distances between residues 241-329 in Nqo12. **H)** Dihedral angles of F/W-333 for WT and the F333W[12] variant.

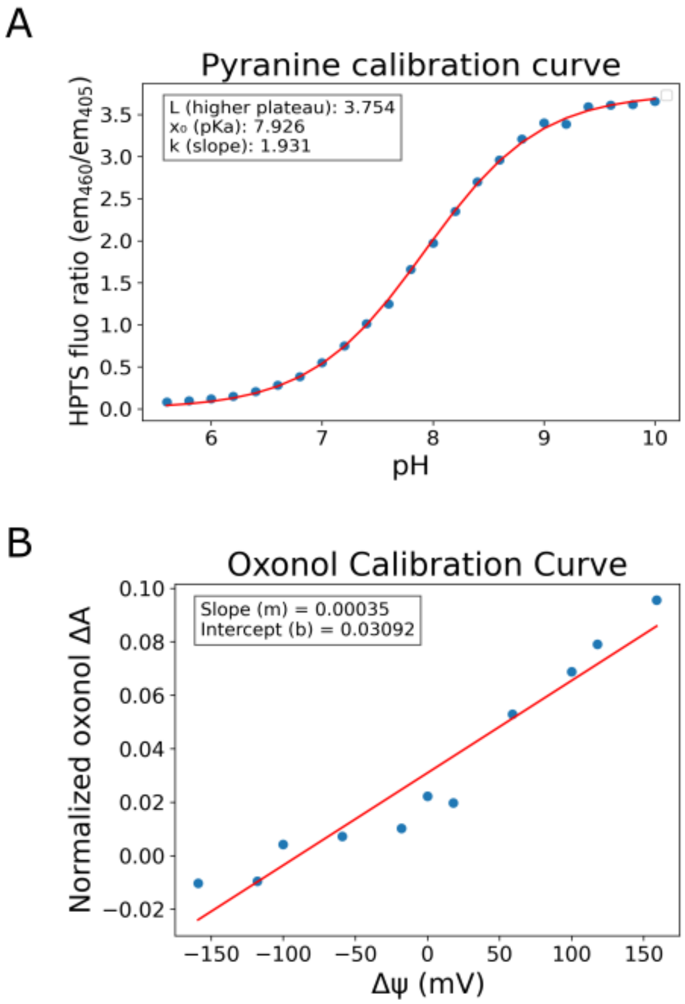

Figure S13. Calibration of pyranine and oxonol VI. A) pH calibration curve for pyranine (HPTS). **B)** Calibration of membrane potential with oxonol VI.

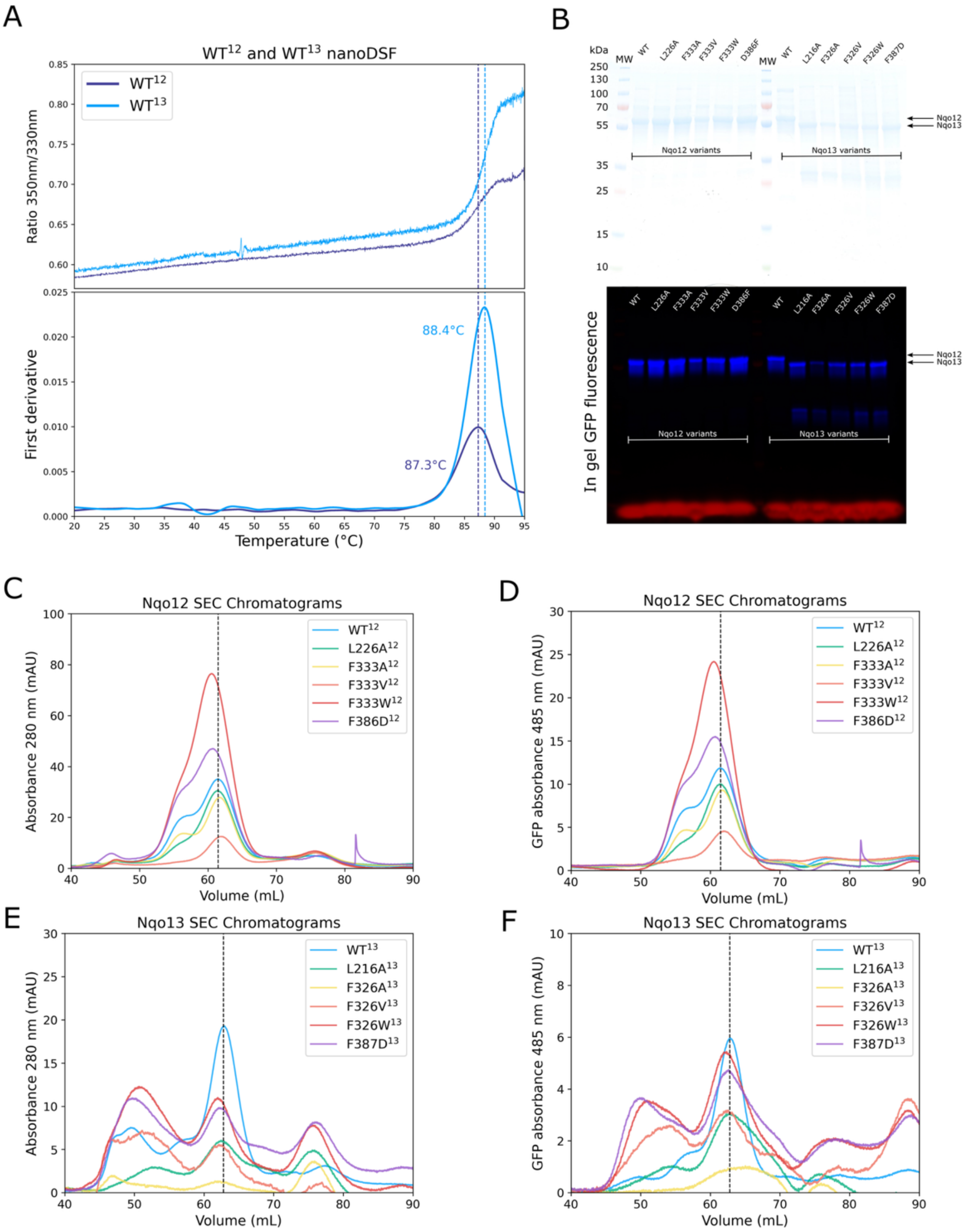

Figure S14. Protein expression, purification, and stability. A) NanoDSF measurements of Nqo12^{WT} and Nqo13^{WT}. **B)** SDS-PAGE gels of Nqo12 and Nqo13 variants with Coomassie staining (*above*) and in-gel fluorescence (*below*). SEC chromatograms of **C-D)**. Nqo12 and **E-F)** Nqo13 chromatograms, showing elution of the different constructs following absorbance at **C,E)** 280 nm and **D,F)** 485 nm.

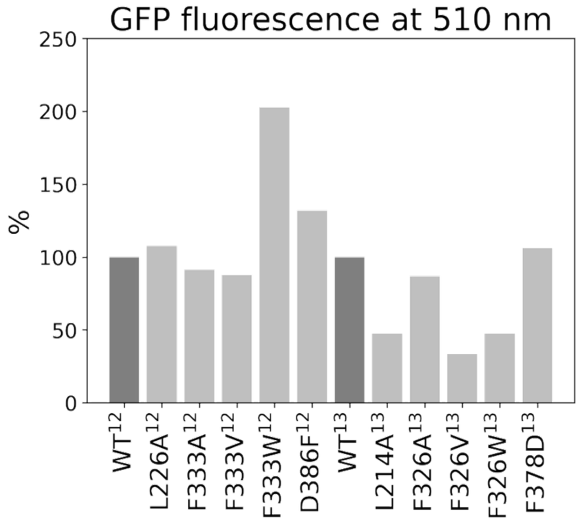

Figure S15. GFP fluorescence of antiporter modules in membranes. GFP signal was scanned between 500 and 600 nm, with excitation at 485 nm. The maximum value at 510 nm in the barplot, is reported for each mutant (grey) relative to the WT (100%, black).

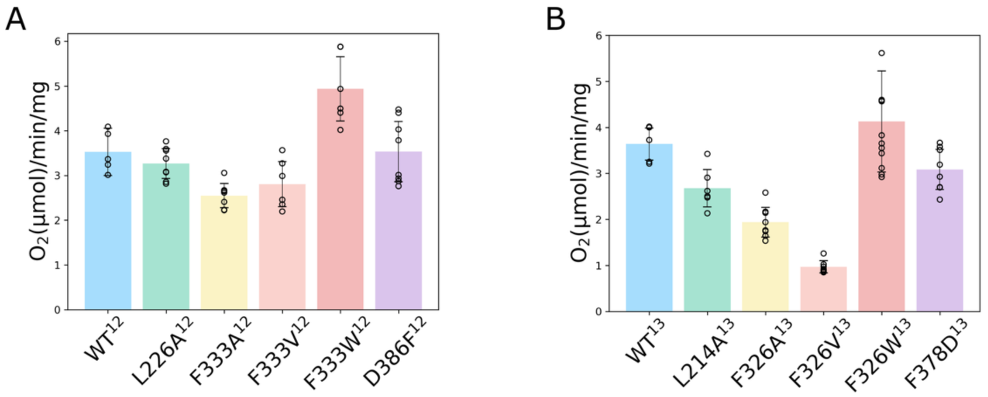

Figure S16. O_2 consumption in membranes upon expression of the antiporter variants. A) Nqo12 and B) Nqo13 oxygen consumption measurements (in µmol of O_2 per minute per milligram of total protein concentration).

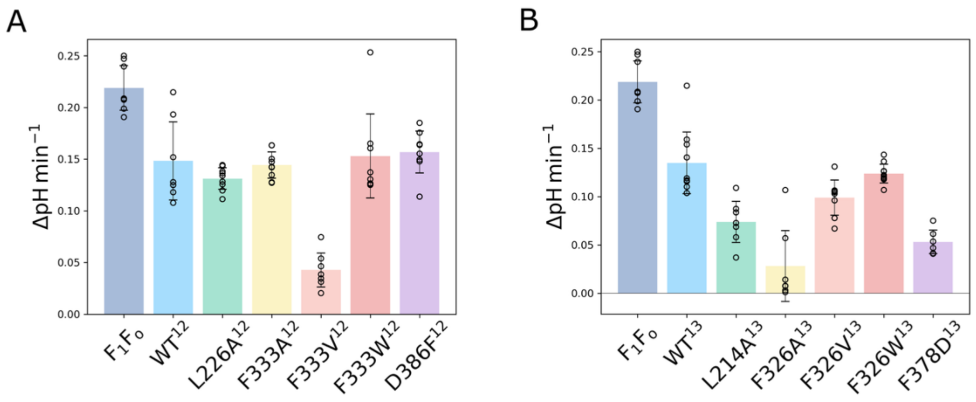

Figure S17. Initial rates of proton conduction from proteoliposome experiments. Initial proton conduction for **A)** Nqo12 and **B)** Nqo13 based proteoliposome experiments with HPTS. The rate was calculated by fitting a linear function to the first 15 seconds of the reaction after ATP addition.

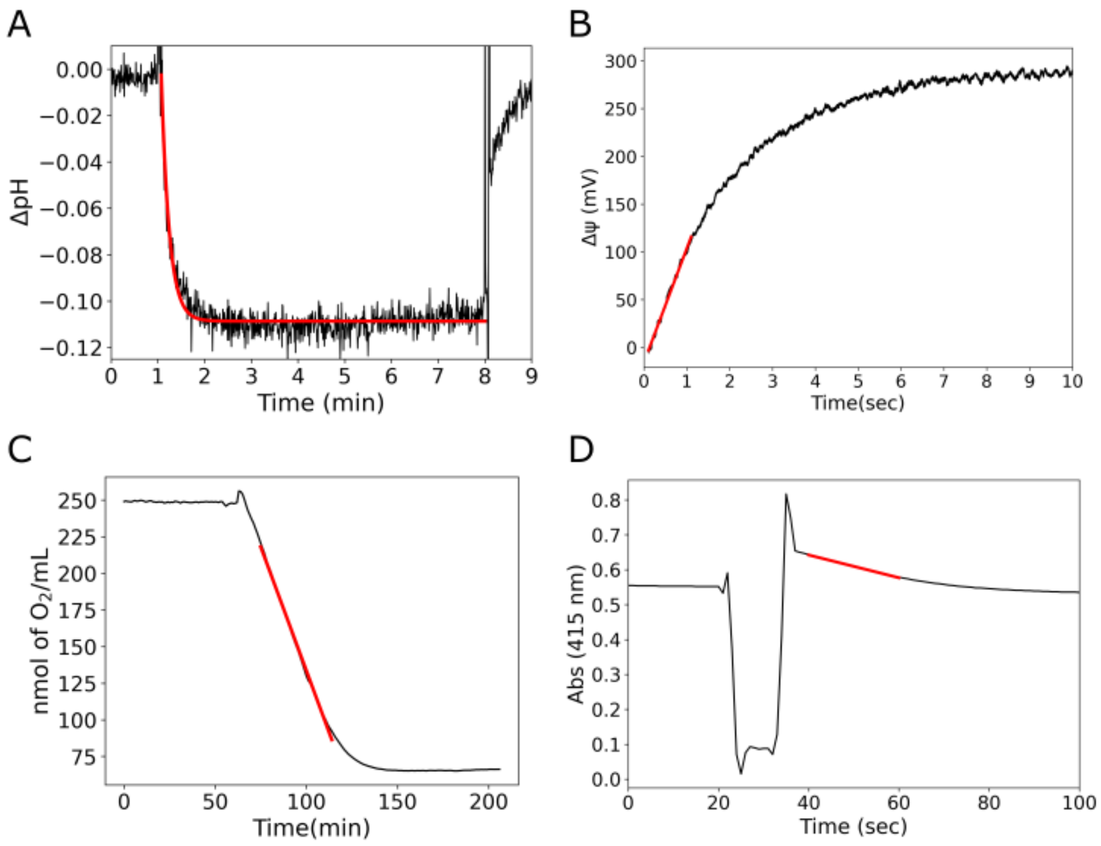

Figure S18. Data fitting. A) Exponential fit of the HPTS measurement. **B)** Linear fit of initial rate from stopped-flow measurement. **C)** Linear fit of O_2 consumption measurement in membranes. **D)** Linear fit of FeCN absorption at 415 nm.

Table S1. List of MD simulations. *p – protonated; n – neutral.*

Simulation	System	Time (ns)	N atoms
**S1**	Nqo13-WT	1000	59,384
**S2**	Nqo13-WT	1000	59,384
**S3**	Nqo13-WT	1000	59,384
**S4**	Nqo13-F326A	1000	59,375
**S5**	Nqo13-F326A	1000	59,375
**S6**	Nqo13-F326A	1000	59,375
**S7**	Nqo13-F326V	1000	59,381
**S8**	Nqo13-F326V	1000	59,381
**S9**	Nqo13-F326V	1000	59,381
**S10**	Nqo13-F326W	1000	59,389
**S11**	Nqo13-F326W	1000	59,389
**S12**	Nqo13-F326W	1000	59,389
**S13**	Nqo13-F378D	1000	59,379
**S14**	Nqo13-F378D	1000	59,379
**S15**	Nqo13-F378D	1000	59,379
**S16**	Nqo13-L214A	1000	59,376
**S17**	Nqo13-L214A	1000	59,376
**S18**	Nqo13-L214A	1000	59,376
**S19**	Nqo12-WT	1000	98,058
**S20**	Nqo12-WT	1000	98,058
**S21**	Nqo12-WT	1000	98,058
**S22**	Nqo12-F333A	1000	98,048
**S23**	Nqo12-F333A	1000	98,048
**S24**	Nqo12-F333A	1000	98,048
**S25**	Nqo12-F333V	1000	98,054
**S26**	Nqo12-F333V	1000	98,054
**S27**	Nqo12-F333V	1000	98,054
**S28**	Nqo12-F333W	1000	98,062
**S29**	Nqo12-F333W	1000	98,062
**S30**	Nqo12-F333W	1000	98,062
**S31**	Nqo12-L226A	1000	98,049
**S32**	Nqo12-L226A	1000	98,049
**S33**	Nqo12-L226A	1000	98,049

**S34**	Nqo12-D386F	1000	98,064
**S35**	Nqo12-D386F	1000	98,064
**S36**	Nqo12-D386F	1000	98,064
**S37**	Nqo12-WT-K329^{n}-H325^{p}	500	98,058
**S38**	Nqo12-WT-K329^{n}-H321^{p}	500	98,058
**S39**	Nqo12-WT-K329^{n}-K385^{p}	500	98,058
**S40**	Nqo12-WT-K329^{n}-D386^{n}	500	98,058
**S41**	Nqo12-WT-K329^{n}	500	98,055
**S42**	Nqo12-WT-K329^{n}-K292^{n}-H241^{p}	500	98,055
**S43**	Nqo13-WT-K235^{n}-H292^{p}	500	59,385
**S44**	Nqo13-WT-K235^{n}-E377^{n}	500	59,385
**S45**	Nqo13-WT-K235^{n}-E306^{n}	500	59,385
**S46**	Nqo13-WT-K235^{n}	500	59,385
**S47**	Nqo13-WT-K235^{n}-K282^{n}-D228^{n}	500	59,386
**S48**	Nqo13-WT-K235^{n}-K282^{n}-H218^{p}	500	59,386

Table S2. List of non-standard protonation states during MD simulations of protonation transfer along the antiporter modules (simulations S37-S48). For the base protonation state (see *Methods*), all residues were modelled in standard protonation states, except $E103^{13}$ (neutral) and $K385^{12}$ (neutral). The histidine residues were modelled δ-protonated, except for H211 H222 H49 and H43, which were modelled Hε-protonated based on prior pK_a calculations[1].

Simulation	System	Protonation states
**S37**	Nqo12-WT-$K329^n$-$H325^p$	$K329^0$, $H325^+$
**S38**	Nqo12-WT-$K329^n$-$H321^p$	$K329^0$, $H321^+$
**S39**	Nqo12-WT-$K329^n$-K385p	$K329^0$, $K385^+$
**S40**	Nqo12-WT-$K329^n$-$D386^n$	$K329^0$, $D386^0$
**S41**	Nqo12-WT-K329n	$K329^+$
**S42**	Nqo12-WT-$K329^n$-$K292^n$-$H241^p$	$K329^0$, $K292^0$, $H241^+$
**S43**	Nqo13-WT-$K235^n$-$H292^p$	$K235^0$, $H292^+$
**S44**	Nqo13-WT-$K235^n$-$E377^n$	$K235^0$, $E377^0$
**S45**	Nqo13-WT-$K235^n$-$E306^n$	$K235^0$ $E306^0$
**S46**	Nqo13-WT-$K235^n$	$K235^0$
**S47**	Nqo13-WT-$K235^n$-$K282^n$-$D228^n$	$K235^0$, $K282^0$, $D228^0$
**S48**	Nqo13-WT-$K235^n$-$K282^n$-$H218^p$	$K235^0$, $K282^0$, $H218^+$

Table S3. List of designed primers.

Oligonucleotide name	Sequence (5' →3')
**Nqo12 TM_L226A_Fwd**	CCCCTCATGGTCTGGGCACCCGACGCCATGGCCGGC
**Nqo12 TM_L226A_Rev**	CCAGACCATGAGGGGGATCTGGG
**Nqo12 TM_F333A_Fwd**	TTCAAGGCCCTCCTCGCACTGGCCTCGGGGAGCGTGATCC
**Nqo12 TM_F333A_Rev**	GAGGAGGGCCTTGAAGAAGGCGTG
**Nqo12 TM_F333V_Fwd**	TTCAAGGCCCTCCTCGTACTGGCCTCGGGGAGCGTGATCC
**Nqo12 TM_F333V_Rev**	GAGGAGGGCCTTGAAGAAGGCGTG
**Nqo12 TM_F333W_Fwd**	TTCAAGGCCCTCCTCTGGCTGGCCTCGGGGAGCGTGATCC
**Nqo12 TM_F333W_Rev**	GAGGAGGGCCTTGAAGAAGGCGTG
**Nqo12 TM_D386F_Fwd**	GGCTTCTGGTCCAAGTTTGCCATCCTCGCCGCCACCC
**Nqo12 TM_D386F_Rev**	CTTGGACCAGAAGCCGGAAAGCAAGG
**Nqo13 L214A_Fwd**	CCGCTGCATGCGTGGGCACCGCCGTTCCACCAAGAGAACCATCC
**Nqo13 L214A_Rev**	CCACGCATGCAGCGGGAACAGC
**Nqo13 F326A_Fwd**	TATACCGGTGGCCTGGCACTGCTGGCGGGCCGTCTGTATGAG
**Nqo13 F326A_Rev**	CAGGCCACCGGTATAAACACCGCTCG
**Nqo13 F326V_Fwd**	TATACCGGTGGCCTGGTCCTGCTGGCGGGCCGTCTGTATGAG
**Nqo13 F326V_Rev**	CAGGCCACCGGTATAAACACCGCTCG
**Nqo13 F326W_Fwd**	TATACCGGTGGCCTGTGGCTGCTGGCGGGCCGTCTGTATGAG
**Nqo13 F326W_Rev**	CAGGCCACCGGTATAAACACCGCTCG
**Nqo13 F378D_Fwd**	GGTTTTCCGGGTGAAGATCTGACCCTGCTGGGTGCGTATAAAGCG
**Nqo13 F378D_Rev**	TTCACCCGGAAAACCGCTCAGGCC

Table S4. K^+_{in}/K^+_{out} concentrations employed for calibration of $\Delta\psi$.

**[K^+_{in}]**	**[K^+_{out}]**	**$\Delta\psi$** =-59 mV log([K^+_{in}]/K[$^+_{out}$])
0.1 mM	50 mM	+159 mV
0.5 mM	50 mM	+118 mV
1 mM	50 mM	+100 mV
5 mM	50 mM	+59 mV
25 mM	50 mM	+18 mV
50 mM	50 mM	0 mV
50 mM	25 mM	-18 mV
50 mM	5 mM	-50 mV
50 mM	1 mM	-100 mV
50 mM	0.5 mM	-118 mV
50 mM	0.1 mM	-159 mV

SI References

1. Beghiah, A.; Saura, P.; Badolato, S.; Kim, H.; Zipf, J.; Auman, D.; Gamiz-Hernandez, A. P.; Berg, J.; Kemp, G.; Kaila, V. R. I. Dissected Antiporter Modules Establish Minimal Proton-Conduction Elements of the Respiratory Complex I. *Nat. Commun.* **2024**, *15* (1), 9098. https://doi.org/10.1038/s41467-024-53194-5.
